# Directed Evolution of an Efficient Polycarbonate Depolymerase With Exceptional Operational Stability

**DOI:** 10.1002/anie.202525215

**Published:** 2026-03-23

**Authors:** Henry A. Jones, Amy E. Hutton, Dominic Harris‐Jukes, John Davidson, Linus O. Johannissen, Colin W. Levy, Michael P. Shaver, Anthony P. Green

**Affiliations:** ^1^ Manchester Institute of Biotechnology, School of Chemistry The University of Manchester Manchester UK; ^2^ Disyn Biotec Ltd. Manchester UK; ^3^ Sustainable Materials Innovation Hub, Department of Materials University of Manchester Manchester UK

**Keywords:** biocatalysis, depolymerization, directed evolution, polycarbonate, polymer hydrolase

## Abstract

We recently developed a high‐throughput directed evolution platform for engineering polymer degrading enzymes, and showcased its utility through the development of an efficient and thermostable variant of *Is*PETase, termed HotPETase. Here, we show that this platform can be used to re‐engineer PET degrading enzymes for the recycling of other aromatic‐containing commodity polymers. Promiscuous poly(bisphenol‐A carbonate) (PC) depolymerase activity of LCC^ICCG^ was enhanced by directed evolution to afford an engineered polycarbonate hydrolase, that also benefits from improved solvent tolerance and operational stability at elevated temperatures. Interestingly, the enzyme‐concentration dependent inhibition observed with the parent enzyme is also alleviated through evolution, improving practical utility. PC‐2 can achieve rapid and complete depolymerization of a PC film to bisphenol‐A (BPA) in just 6 h at 75°C. This study shows how plastic degrading enzymes can be readily adapted through evolution to operate on new and valuable polymer classes.

## Graphical abstract

Here, we show that well studied PET hydrolases can be re‐engineered via directed evolution to deconstruct alternative aromatic‐containing commodity polymers. Introduction of only three mutations into LCC^ICCG^ was sufficient to generate an efficient polycarbonate hydrolase with high operational stability that can fully depolymerize a polycarbonate sample within 6 h at 75°C.

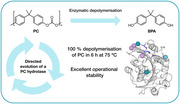

## 1 Introduction

Growing global demand for plastic products, coupled with their resistance to degradation at end‐of‐life, poses a significant environmental and economic challenge [1]. Annual plastic production is projected to more than triple by 2060 [2], necessitating the development of improved recycling technologies and infrastructure to alleviate environmental concerns [3]. For polymers with hydrolysable backbones, depolymerization can offer an attractive recycling strategy, allowing the recovery of constituent monomers and circularization of the plastic life‐cycle [4]. For some synthetic polymers, such as poly(ethylene terephthalate) (PET) and poly(lactic acid) (PLA), enzymatic depolymerization has emerged as an attractive alternative to current chemical recycling methods [5, 6]. Such biocatalytic conversions can operate under relatively mild conditions and offer the potential for selective depolymerization of mixed plastic waste streams [7].

Achieving commercial scale enzymatic depolymerization relies on the development of robust engineered biocatalysts that can operate efficiently under viable process conditions [8, 9, 10, 11]. In the case of PET, engineered depolymerases are now available that meet these demands, enabling commercial scale recycling [12, 13]. This success is now inspiring efforts to discover and engineer biocatalysts for deconstructing alternative polymer materials that are more challenging to mechanically recycle [14], including polyurethanes (PUs) [15, 16], polyamides (PAs) [17], polyhydroxyalkanoates (PHAs) [18], and poly(butylene adipate terephthalate) (PBAT) [19].

In contrast to the structural heterogeneity of industrially produced PUs, PAs, and polyesters, commercial PC is produced almost exclusively as a single polymer, poly(bisphenol‐A carbonate), on a nearly five million metric tons per annum scale [20]. Prized for its structural rigidity, toughness, high impact strength, thermal stability, clarity, and transparency, PC has found widespread use in the automotive, electronics, construction, and medical sectors, among others [21]. The ubiquity of PC in these essential industries is driving increased production, projected at 6.2% per annum to the year 2031 [22]. As such, there is great interest in the development of new end‐of‐life solutions for PC, as mismanaged or landfilled PC waste represents a significant loss of economic value and poses the risk of harmful BPA leaching into the environment [23].

**FIGURE 1:**
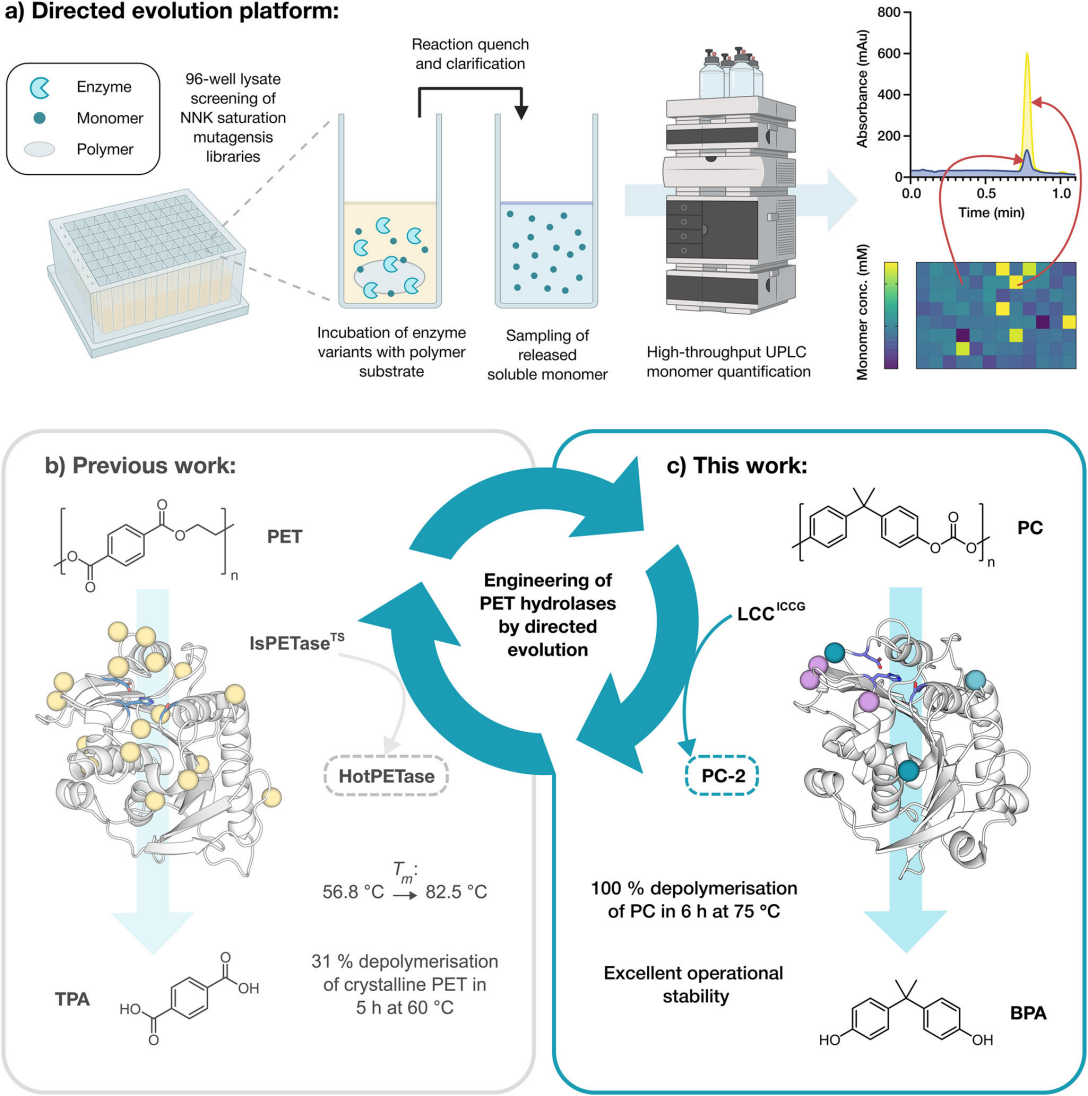
(a) Our directed evolution platform for engineering polymer degrading enzymes, which utilizes a lysate‐based screening approach to rapidly assess numerous NNK saturation mutagenesis libraries for improved depolymerase variants. High‐throughput UPLC analysis of soluble monomer release allows efficient and accurate assessment of variants to identify improvements in activity. An example library plate from the second round of evolution documented is this paper is shown, along with a corresponding UPLC trace of the parent template (blue) overlayed with an improved variant (yellow). (b) A summary of our previous evolution campaign, affording HotPETase, a substantially more active and thermostable variant of *Is*PETase [8]. (c) In this work a PET hydrolase, LCC^ICCG^ [12], has been re‐engineered using our directed evolution platform to efficiently depolymerize PC.

Chemical recycling of PC in the form of hydrolysis, alcoholysis, aminolysis, and hydrogenolysis have been extensively researched [4]. In contrast, there are few reports describing enzymatic PC degradation. Early work focused on the use of commercial lipases to deconstruct PC dissolved in organic solvents, with only modest levels of polymer degradation observed [24, 25]. During preparation of this manuscript, Schell et al. reported the depolymerization of PC using known cutinases, with the data presented in accordance with some of the early findings from this study [20]. Finally, Holst et al. detailed the *de novo* design of a PC hydrolase, mdArmRP, utilizing Rosetta to install a surface exposed catalytic triad into a thermostable scaffold [26]. Activity of the resulting enzyme (*T_m_* > 95°C) was evidenced by *µ*m changes in the PC substrate surface as monitored by atomic force microscopy. In this study we establish a directed evolution pipeline that, allows quantitative analysis of >1,000 polycarbonate hydrolase variants per day (Figure 1). This platform was used to engineer a proficient polycarbonate hydrolase with high operational stability.

## 2 Results and Discussion

To identify a suitable starting template for PC hydrolase engineering, we evaluated a panel of previously reported PET hydrolases for promiscuous PC depolymerase activity. The recently reported designed PC hydrolase, mdArmRP, was also included in this panel [26]. Activity was assessed using an ultra‐high performance liquid chromatography (UPLC) assay to quantify BPA monomer formation following incubation of a 3 mm amorphous PC disc (Goodfellow 0.25 mm film, henceforth referred to as *Gf*‐PC discs) with purified enzymes. Biotransformations were performed over a range of temperatures (45–70° C), spanning the previously reported temperature optima for PET deconstruction, and under three buffer conditions commonly used for enzymatic PET depolymerization.

**FIGURE 2:**
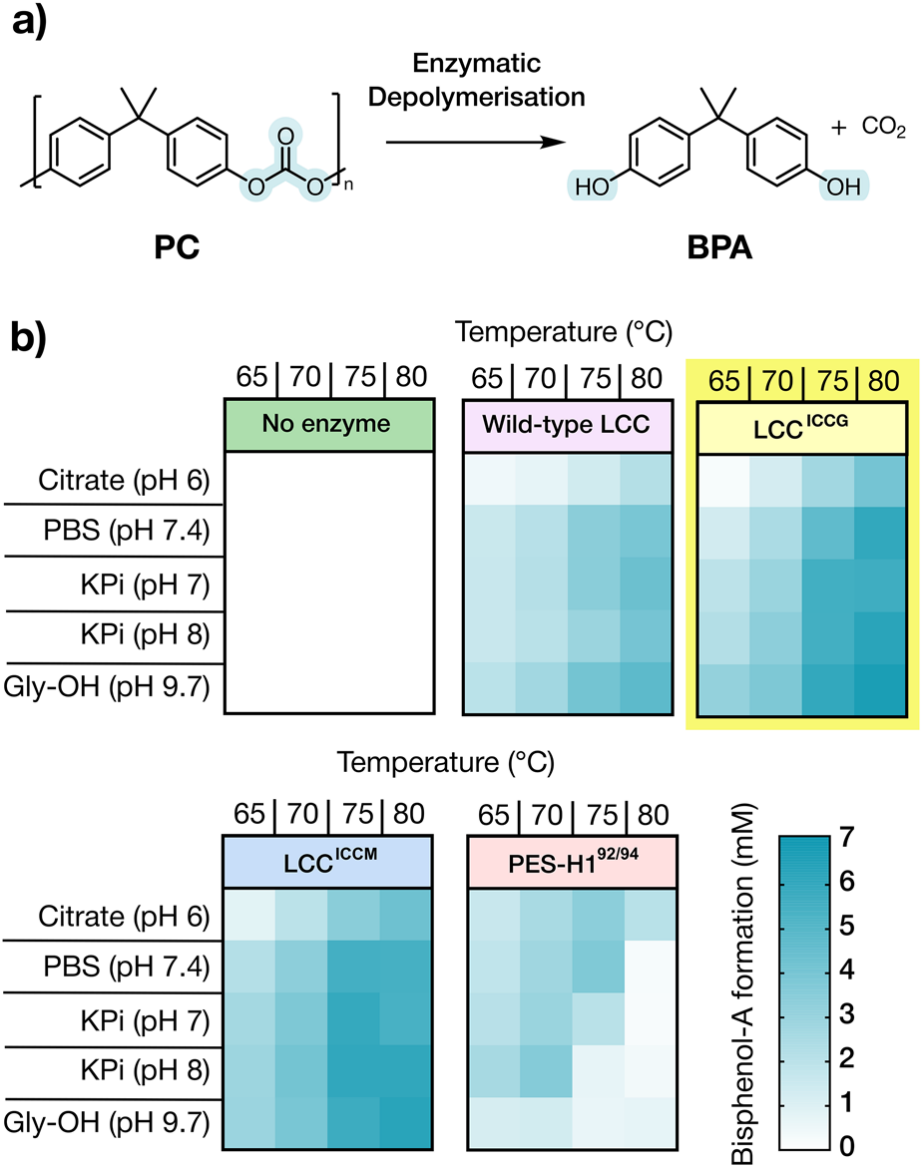
(a) Target transformation for the depolymerization of PC to monomeric BPA. (b) Temperature‐activity profiling of the mean total concentration of BPA produced from PC depolymerization by a curated panel of cutinases over a range of buffer conditions. Biotransformations were performed as 100 *µ*L reactions with a *Gf*‐PC disc, enzyme (0.5 *µ*M), 4% (v/v) BugBuster, 20 h incubation at 850 r.p.m. The heatmap gradient represents the released BPA monomer equivalents from 0 to 7 mM with each square representing the average of reactions carried out in triplicate.

Of the 20 enzymes tested, 18 gave detectable BPA formation under at least one of the temperature and buffer conditions investigated (Figure S1). Engineered *Is*PETase variants, the carboxyl esterase CE‐Ubrb, and the designed mdArmRP displayed modest activity (<1 mM BPA), while no detectable monomer release was observed for cutinases HiCut and Cut190*. In contrast, 9 cutinases were found to generate >1 mM BPA following 20 h incubation. Based on these initial assays we selected four enzymes (wild‐type LCC, LCC^ICCG^, LCC^ICCM^, and PES‐H1^92/94^) for further evaluation across a broader range of reaction conditions including temperatures (65–80°C) and buffers (pH 6–9.7) (Figure 2) [12, 27, 28]. Following this screening, we selected LCC^ICCG^ as a template for evolutionary optimization. This variant performed optimally at 80°C in a glycine buffer at pH 9.7, affording 6.73 mM of BPA monomer using 0.5 *µ*M enzyme (>10,000 turnovers).

**FIGURE 3:**
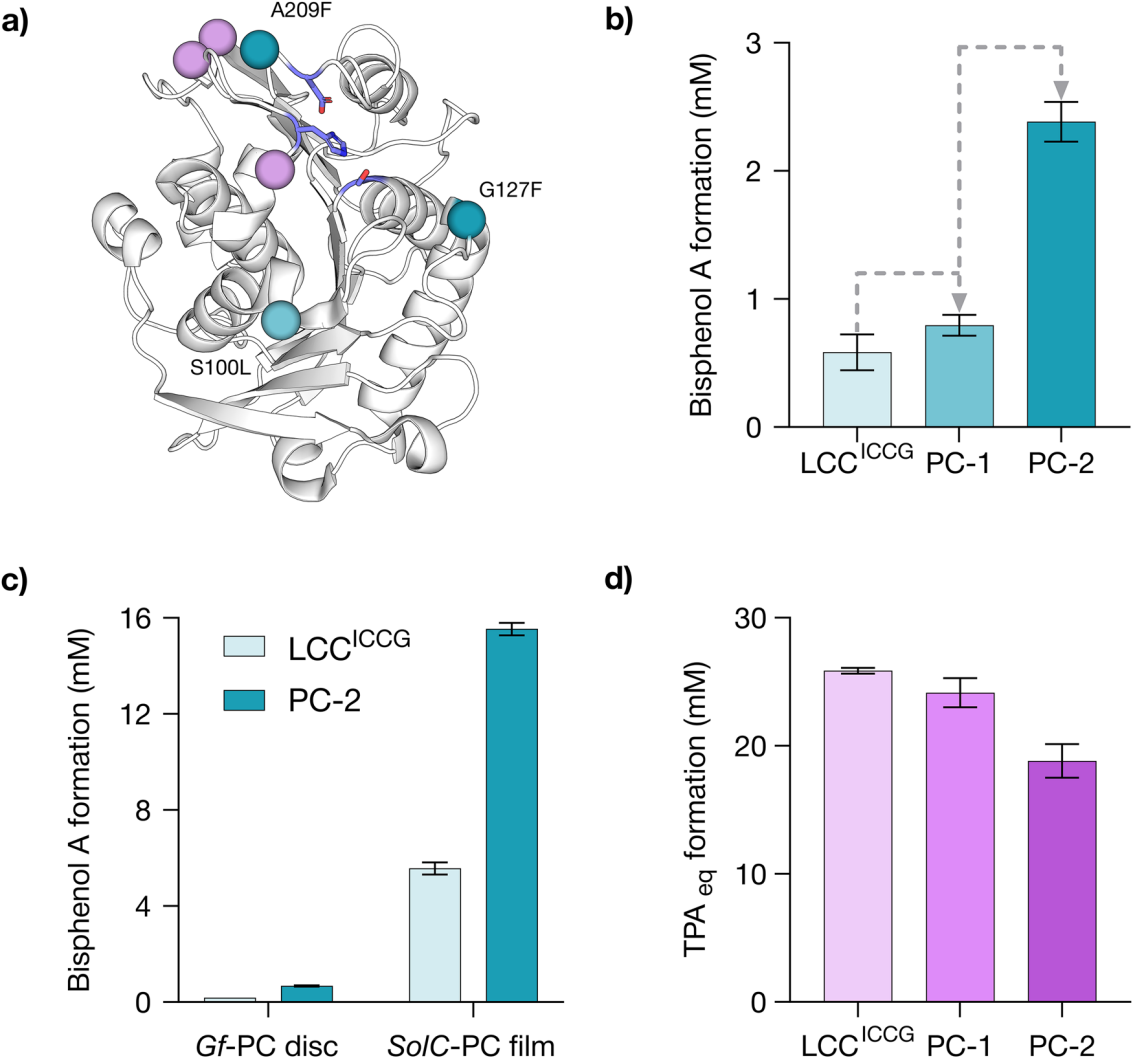
(a) Structure showing the amino acid positions mutated in PC‐2 mapped onto the crystal structure of LCC^ICCG^ (PDB: 8OTA) [30]. Mutations are represented as spheres at the C_α_, with blue spheres corresponding to those introduced during evolution (Figure 3b), and purple spheres corresponding to LCC^ICC^. The Ser165‐His242‐Asp210 catalytic triad is shown as atom‐colored sticks with blue carbons. (b) Bar chart showing improvements in the mean total concentration of BPA produced along the evolutionary trajectory under the evolutionary assay conditions. Biotransformations were performed as 100 *µ*L reactions with a single *Gf*‐PC disc, enzyme (5 *µ*M) in Gly‐OH (pH 9.7, 50 mM) supplemented with 4% (v/v) BugBuster, 8 h at 65°C. Error bars represent the standard deviation of measurements made in triplicate. (c) Bar chart showing activity improvements observed when changing substrate from *Gf*‐PC discs to *SolC*‐PC films. Biotransformations were performed as 100 *µ*L reactions with a single *Gf*‐PC disc or single *SolC*‐PC film, enzyme (5 *µ*M) in Gly‐OH (pH 9.7, 50 mM) supplemented with 4% (v/v) BugBuster, 2 h at 65°C. Error bars represent the standard deviation of measurements made in triplicate. (d) Bar chart showing the decrease in PET depolymerization (monitored by summative formation of terephthalic acid (TPA) and mono(2‐hydroxyethyl) terephthalate (MHET), TPA_eq_) observed along the evolutionary trajectory under the evolution assay conditions. Biotransformations were performed as 100 *µ*L reactions with a single *Gf*‐PET disc, depolymerase (5 *µ*M) in Gly‐OH (pH 9.7, 50 mM) supplemented with 4% (v/v) BugBuster, 2 h at 65°C. Error bars represent the standard deviation of measurements made in triplicate.

To improve activity, LCC^ICCG^ was subjected to sequential rounds of laboratory evolution utilizing our previously developed evolution platform for engineering polymer degrading enzymes [8]. Individual library variants were arrayed in 96‐well plates and evaluated as clarified cell lysates, using a UPLC assay to monitor BPA release from *Gf*‐PC discs. Assays were performed at 65°C due to constraints of the operational temperature range of the incubators used. The evolutionary strategy employed two rounds of saturation mutagenesis, using degenerate NNK codons to individually randomize 22 residue positions per round (Table S1). The most active (*ca*. 1%) clones from each round were selected for further evaluation as purified proteins. Beneficial mutations identified in each round were subsequently combined by DNA shuffling. After evaluation of >3,800 clones a variant, PC‐2, emerged that contained three mutations (S100L, G127F, and A209F). Under the assay conditions used for evolution, PC‐2 gave a 4.1‐fold increase in BPA release after 8 h (Figure 3a,b), with more pronounced improvements observed following optimization of assay conditions (see below). These activity improvements also correlate to a ca. 2‐fold improvement in activity toward a model BPA‐dimer, both in terms of initial rate and total turnover number (Figure S2). Interestingly, evolution also alleviated enzyme concentration dependent inhibition behavior (Figure S3), observed with LCC^ICCG^ during PC deconstruction, a phenomenon previously reported for mesophilic PET depolymerases [29]. In contrast to the observed improvements in PC deconstruction following evolution, PET depolymerase activity was reduced by ca. 30% in PC‐2 compared to LCC^ICCG^, while activity toward a model bis(2‐hydroxyethyl) terephthalate (BHET) substrate is essentially unaffected (Figures 3d and S2). Although at an early stage of evolution, these observations hint at the potential to develop selective depolymerases for PC versus PET deconstruction.

To maximize the extent of PC depolymerization, we optimized assay conditions, exploring a range of parameters including substrate morphology, temperature, enzyme loading, and buffer/cosolvent composition. As well as commercial films, micronized powders are often utilized in enzymatic depolymerization studies as a suitably uniform substrate with a substantially increased surface area. To this end, a commercial PC powder was trialed as an alternate substrate, however substantial residual BPA present in the purchased powder, coupled with extensive electrostatic adhesion of the fine powder to glassware and plasticware, impeded safe, and accurate handling. As such, a method of 96‐well PC film solvent‐casting was developed to give a safe and operationally convenient substrate with an increased surface area (henceforth, referred to as *SolC*‐PC). We confirmed that the polymer molecular weight, dispersity, and crystallinity were not affected by the casting process (Figures S4 and S5).

**FIGURE 4:**
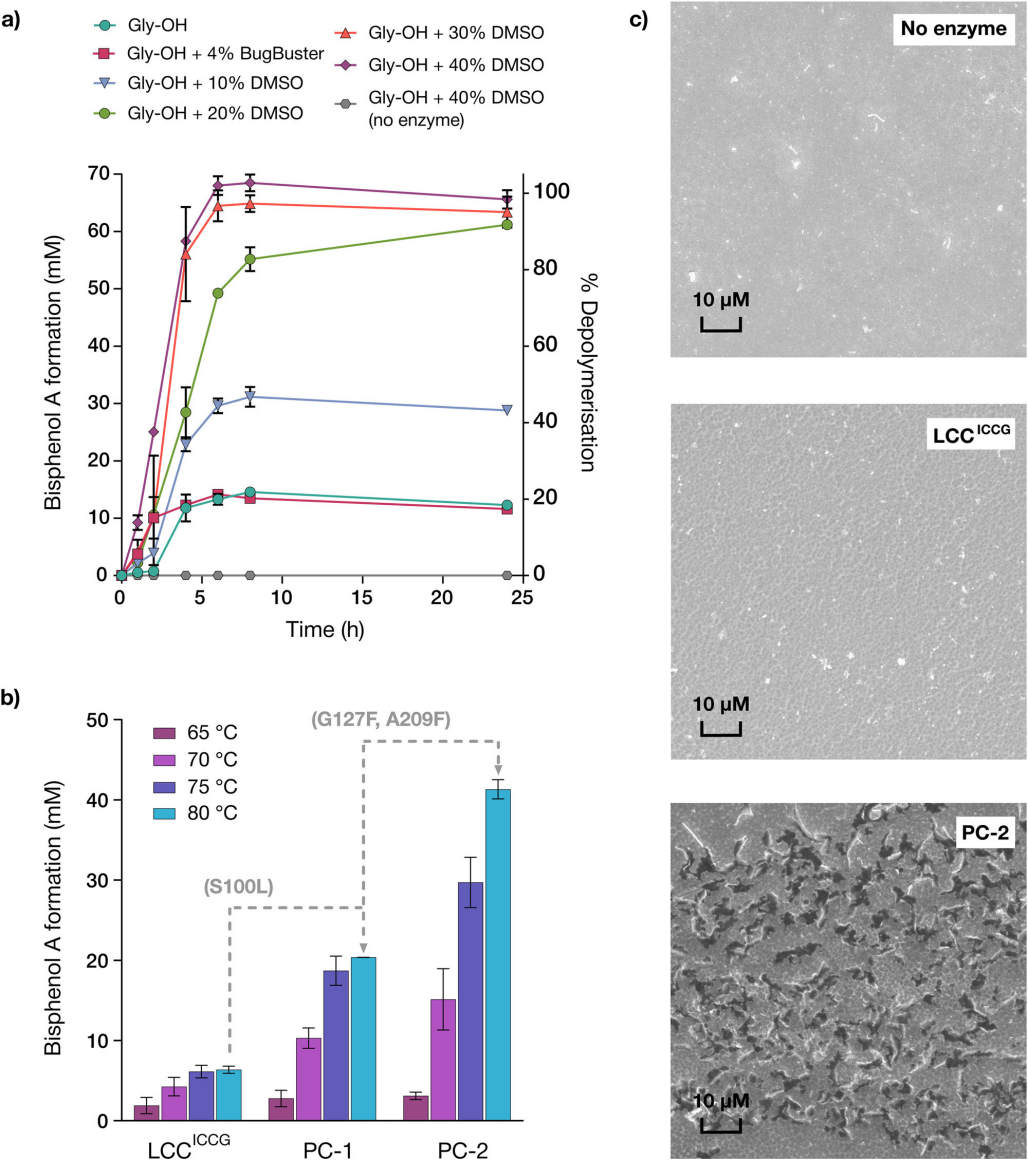
(a) 24 h time‐course reactions of *SolC*‐PC deconstruction by PC‐2 under varying DMSO cosolvent loadings, showing the mean total concentration of produced BPA (left y axis) and the mean percentage substrate depolymerization (right y axis). Biotransformations were performed as 100 *µ*L reactions with a single *SolC*‐PC film, PC‐2 (5 *µ*M) in Gly‐OH (pH 9.7, 50 mM) supplemented with varying DMSO cosolvent loadings (0%–40%), 65° C, 850 r.p.m. Error bars represent the standard deviation of measurements made in triplicate. (b) Bar chart showing the mean total concentration of BPA produced from *SolC*‐PC deconstruction along the evolutionary trajectory at elevated temperatures under optimized conditions. Biotransformations were performed as 100 *µ*L reactions with a single *SolC*‐PC film, enzyme (10 *µ*M) in Gly‐OH (pH 9.7, 50 mM), 40% DMSO, 1 h at 850 r.p.m. in an Eppendorf Thermomixer C tabletop plate shaker. Error bars represent the standard deviation of measurements made in triplicate. (c) SEM images of *SolC*‐PC film following 2 h incubation at 65°C with no enzyme (top), 5 *µ*M LCC^ICCG^ (middle), or 5 *µ*M PC‐2 (bottom). Biotransformations were performed as 100 *µ*L reactions with a single *SolC*‐PC film in Gly‐OH (pH 9.7, 50 mM), 40% DMSO, 2 h, 850 r.p.m. at 65° C.

This modified substrate led to a >20‐fold increase in the rate of depolymerization, with evolutionary improvements found with *Gf*‐PC discs also observed with *SolC*‐PC (Figure 3c). As the commercial film is more ordered through controlled film extrusion and annealing, and may also be coated to improve dimensional stability, this may limit enzyme access to polymer chains, limiting observed rates. Solvent casting would remove both possible barriers. Using *SolC*‐PC, a reaction time course of depolymerization by PC‐2 (5 *µ*M) shows fast formation of BPA monomer (∼15.5 mM), corresponding to a ∼25% depolymerization) over the first 2 h, after which time the reaction stalls (Figure S6). Addition of fresh enzyme or increasing buffering capacity failed to increase monomer formation (Figure S7), suggesting that neither enzyme deactivation nor pH changes during biotransformation is responsible for incomplete depolymerization. In contrast, subjecting partially degraded *SolC*‐PC to fresh buffer and enzyme led to a comparable reaction profile as that observed with pristine substrate (Figure S7). These observations are consistent with an apparent product‐dependent inhibition, potentially linked to the solubility limit of BPA in buffer (for example, precipitated monomer could coat the polymer surface and hinder further enzymatic deconstruction). To explore this hypothesis, we investigated the use of DMSO as a reaction cosolvent (Figure 4a). Pleasingly, with 40% DMSO, PC‐2 achieved complete depolymerization within 8 h with an accompanying increase in the initial rate of depolymerization. While the benefit of DMSO was also reported for LCC^ICCG^ as we were preparing this manuscript [20], LCC^ICCG^ was unable to achieve full depolymerization even after 24 h incubation. The beneficial effect of DMSO has been ascribed to swelling of the polymer structure to facilitate enzymatic attack [20]. This optimized reaction protocol could be further improved by using PC‐2 at elevated temperatures, with complete depolymerization achieved within 6 h at 75°C (equating to ∼7,000 turnovers) (Figure S8). We note that at reduced PC‐2 loading (1 *µ*M), the enzyme can achieve ∼60,000 turnovers prior to deactivation, with a specific activity of 1,071 *µ*mol mg^−1^ h^−1^ (Figure S8). A substantial improvement in reaction rate was also observed upon increasing reaction temperature from 65 to 80°C, with 41.3 mM BPA formed within 1 h (>50% depolymerization) using 10 *µ*M enzyme (Figures 4b,S9). Notably, despite enzyme engineering being performed at 65°C, these temperature dependent activity gains are more pronounced for PC‐2 compared with LCC^ICCG^ suggesting the operational stability of the enzyme has been enhanced following evolution. The improved activity of PC‐2 compared to LCC^ICCG^ was also supported by scanning electron microscopy images, which visually evidence the more rapid degradation of *SolC*‐PC films (Figure 4c) [30]. We also compared the activity of PC‐2 to ThcCut1^ACCG^ which was recently shown to have comparable PC hydrolase activity to LCC^ICCG^. As anticipated, PC‐2 outperforms ThcCut1^ACCG^ across a range of reaction conditions and PC morphologies (Figure S10).

**FIGURE 5:**
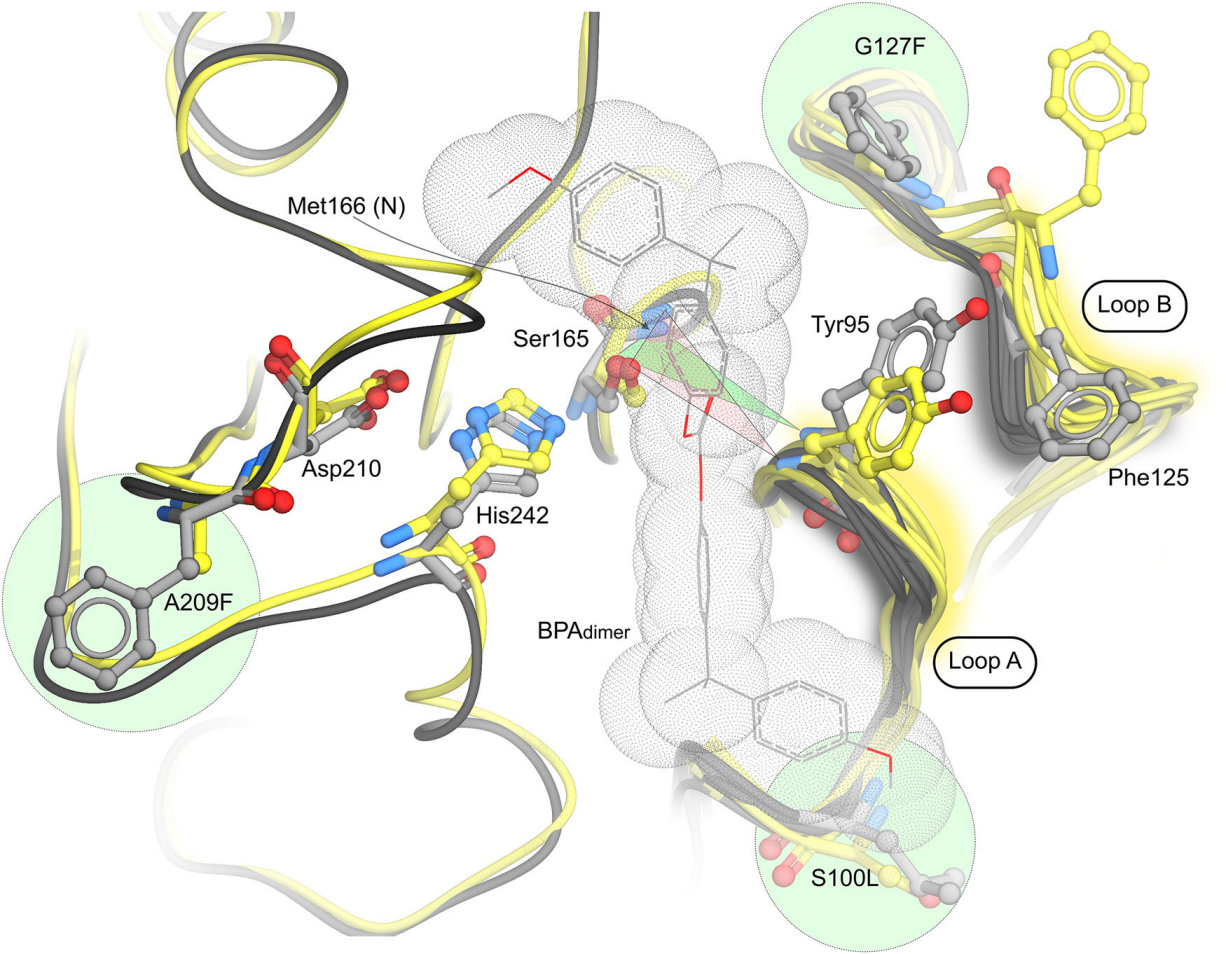
A protein worm representation of selected MD frames of PC‐2 (grey) and LCC^ICCG^ (yellow). For each protein, eight frames, corresponding to 100 ns snapshots along the MD trajectory were superimposed in ICM Pro. For clarity, complete backbone traces are shown for single chains of both the PC‐2 and LCC^ICCG^ whilst all backbone traces are shown in the loop A and B regions. The three installed mutations, S100L, G127F, and A209F are highlighted by green circles and displayed in all atom colored ball and stick. The position of the docked BPA dimer is shown in stick representation with accompanying dot surfaces. A green triangle highlights the position of the catalytic serine, S165, and oxyanion hole in PC‐2 whilst a red triangle marks similar features in LCC^ICCG^.

To gain insights into the origins of enhanced operational stability and PC hydrolysis activity following directed evolution, we performed MD simulations on LCC^ICCG^ and an AlphaFold2 model of PC‐2. These calculations reveal that two loops (94‐97 (loop A) and 121–125 (loop B)) are substantially less mobile in PC‐2. The G127F mutation narrows the distribution and increases the average 125_cα_126_cα_‐127_cα_128_cα_ dihedral angle and shortens the distance between loops A and B (Figure S11). The changes in loop A conformation result in a narrower active site cleft and alter the geometry of the oxyanion hole (NH backbone of Tyr95 and Met166). Docking of a BPA dimer into a representative frame from the PC‐2 simulations suggest that, these structural changes can help to promote ligand binding and positioning into a productive pose for catalysis (Figure 5).

## 3 Conclusion

Enzymatic polymer deconstruction has emerged as an attractive alternative to current chemical plastic recycling. A significant proportion of these efforts have focused on PET deconstruction with engineered depolymerases now available for commercial scale recycling. Here, we show that a commonly employed PET hydrolase can be readily adapted by laboratory evolution to efficiently operate on other abundant classes of hydrolysable polymer. Following evaluation of a diverse panel of PET hydrolases we identified LCC^ICCG^ as a promising candidate for PC hydrolase engineering. Interestingly despite a high degree of structural homology to LCC, engineered thermostable variants of *Is*PETase were found to be only poorly active on PC substrates. The polycarbonate hydrolase activity of LCC^ICCG^ was subsequently optimized by directed evolution, capitalizing on our recently established pipeline for engineering polymer degrading enzymes. Introduction of only three mutations was sufficient to generate an efficient PC hydrolase with high operational stability that can fully depolymerize a polycarbonate sample within 6 h at 75° C. We anticipate that more extensive laboratory evolution will deliver engineered variants with further enhanced properties. Moving forward, we are optimistic that directed evolution of natural and/or designed enzymes will deliver biocatalysts for selectively deconstructing a wide range of polymer materials to recover value at end‐of‐life.

## Conflicts of Interest

The authors declare no conflicts of interest.

## Supporting information



The authors have cited additional references within the Supporting Information [31, 32, 33, 34, 35, 36, 37, 38, 39, 40, 41, 42, 43, 44, 45, 46, 47].
**Supporting File**: anie71931‐sup‐0001‐SuppMat.docx.

## Data Availability

The data that support the findings of this study are available from the corresponding author upon reasonable request.
