# Supplementary material for: Directed Evolution of an Efficient Polycarbonate Depolymerase With Exceptional Operational Stability
Source: Angew Chem Int Ed Engl. 2026 Mar 23;65(18):e25215. doi: 10.1002/anie.202525215 (PMC13110763; doi:10.1002/anie.202525215)
Supplement: Supplementary file 1 — The authors have cited additional references within the Supporting Information [31, 32, 33, 34, 35, 36, 37, 38, 39, 40, 41, 42, 43, 44, 45, 46, 47]. Supporting File: anie71931‐sup‐0001‐SuppMat.docx. [file ANIE-65-e25215-s001.docx]

Supporting information for

Directed Evolution of an Efficient Polycarbonate Depolymerase with Exceptional Operational Stability

Henry A. Jones^[a]^, Amy E. Hutton^[a,b]^, Dominic Harris-Jukes^[c]^, John Davidson^[a]^, Linus O. Johannissen^[a]^, Colin W. Levy^[a]^, Michael P. Shaver^[c]*^, Anthony P. Green^[a]*^

[a] H. A. Jones, A. E. Hutton, J. Davidson, A. P. Green
Manchester Institute of Biotechnology
School of Chemistry
131 Princess Street, The University of Manchester, Manchester M1 7DN, UK
E-mail: [anthony.green@manchester.ac.uk](mailto:anthony.green@manchester.ac.uk)

[b] A. E. Hutton
Disyn Biotec Ltd.
131 Princess Street, Manchester M1 7DN, UK

[c] D. Harris-Jukes, M. P. Shaver
Sustainable Materials Innovation Hub
Department of Materials
University of Manchester, Oxford Road, Manchester, M13 9PL, UK
E-mail: [michael.shaver@manchester.ac.uk](mailto:michael.shaver@manchester.ac.uk)

**Contents**

[Materials and methods 2](#_Toc213428752)

[Supplementary Figures 9](#_Toc213428753)

[Supplementary Tables 26](#_Toc213428754)

[DNA and Protein Sequences 32](#_Toc213428755)

[References 34](#_Toc213428756)

# **Materials and methods**

**Materials**

All chemical and biological materials were purchased from commercial suppliers. Kanamycin, ampicillin, DNase I, bisphenol-A (BPA), Bis(2-hydroxyethyl) terephthalate (BHET) and terephthalic acid (TPA) were obtained from Sigma-Aldrich; 4-[1-(4-methoxyphenyl)-1-methylethyl]phenol was obtained from Apollo Scientific; LB agar, LB media, 2 x YT media, and arabinose from Formedium; BugBuster® Protein Extraction Reagent from Merck; *E. coli* 5-alpha, *E. coli* BL21 (DE3), Q5 DNA polymerase, T4 DNA ligase, and restriction enzymes from New England BioLabs; *Escherichia coli* Origami 2 (DEQ) from Novagen; 0.25 mm thickness amorphous poly(ethylene terephthalate) (PET) sheet (*Gf*-PET, catalogue number: GF54024997) and 0.25 mm thickness amorphous PC Lexan 8010 film from Goodfellow (*Gf*-PC); PC pellets (Makrolon 2407) from Plastiserve; PC powder (<300 micron particle size, catalogue number: NCZ-LP-112/23) from Nanochemazone, and oligonucleotides were synthesized by Integrated DNA Technologies.

**Gene constructions**

Genes encoding *Is*PETase^TS^,^1^ M6, M9, M10, HotPETase,^2^ TfCut2,^3^ and CE-Ubrb^4^ were cloned into the *NdeI* (5′ end) and *XhoI* (3′ end) sites of a pBbE8K vector modified to contain a *C*-terminal hexa-histidine tag coding sequence following the *XhoI* restriction site.^5^ Genes encoding HiCut^6^ and Cut190*^7^ were cloned into the NdeI (5′ end) and XhoI (3′ end) sites of pET-26b vector (Novagen) leading to fusion to a *C*-terminal hexa-histidine tag coding sequence. Genes encoding LCC,^8^ LCC^ICCG^, LCC^ICCM^, LCC^WCCG^, LCC^WCCM^,^9^ PES-H1, PES-H1^92/94^, PES-H1^204/250^,^10^ FASTPETase,^11^ DuraPETase,^12^ and mdArmRP^13^ were cloned into a pET26-ccdB vector using *BsaI*-HFv2, employing the golden gate assembly method leading to fusion to a *C*-terminal hexa-histidine tag coding sequence.^14^ Genes were codon optimised for expression in *Escherichia coli*. Nucleotide sequences encoding signal peptides were removed prior to cloning as described in the literature.^2,9^

**Production of purified proteins**

*Is*PETase^TS^ and its derivatives were expressed in chemically competent Origami 2 *E. coli*. Single colonies of freshly transformed cells were cultured (18 h at 37 °C, 200 r.p.m.) in LB medium (5 mL) supplemented with kanamycin (25 *µ*g mL^−1^) and tetracycline (2.5 *µ*g mL^−1^). Starter cultures (1 mL) were used to inoculate 2YT medium (50 mL) containing kanamycin (25 *µ*g mL^−1^) and tetracycline (2.5 *µ*g mL^−1^). Cultures were grown (35 °C, 180 r.p.m.) to an OD_600_ of 1.0. Protein production was initiated by the addition of L-arabinose (final concentration of 10 mM) and cultures were incubated for 20 h (19 °C, 180 r.p.m.). Cells were collected by centrifugation (2,900 x *g* for 10 min). Cells were resuspended in lysis buffer (pH 7.5, 50 mM Tris-HCl, 10 mM imidazole, 300 mM NaCl, 10 *µ*g mL^−1^ DNase I). Cells were disrupted by sonication and the resulting lysate clarified by centrifugation (10,000 x *g* for 15 min). The soluble fraction was subjected to affinity chromatography using Ni-NTA agarose (Qiagen). Unbound proteins were washed off with lysis buffer supplemented with 10 mM imidazole, and bound proteins were eluted with elution buffer (pH 7.5, 50 mM Tris-HCl, 300 mM imidazole, 300 mM NaCl). Proteins were desalted by application to 10DG desalting columns (Bio-Rad) and eluted in storage buffer (pH 7.5, 50 mM Tris-HCl, 150 mM NaCl).

TfCut2, and CE-Ubrb were expressed in chemically competent *E. coli.* BL21 (DE3), and cultured and purified as above.

DuraPETase and FASTPETase were expressed in chemically competent *E. coli*. C43 (DE3), and cultured and purified as above.

LCC and its derivatives, PES-H1 and its derivatives, Cut190*, HiCut, ThcCut1^ACCG^ and mdArmRP were expressed in chemically competent *E. coli.* BL21 (DE3). Single colonies of freshly transformed cells were cultured (18 h at 37 °C, 200 r.p.m.) in LB medium (5 mL) supplemented with kanamycin (50 *µ*g mL^−1^). Starter cultures (1 mL) were used to inoculate auto-inducible ZYM medium (100 ml) containing kanamycin (50 *µ*g mL^−1^).^15^ Cultures were incubated for 22 h (21 °C, 180 r.p.m.). Cells were subsequently collected by centrifugation (2,900 x *g* for 10 min) and purified as above.

**Library construction**

In each round of evolution, 22 positions were individually randomized using primers with NNK degenerate codons (Table S2). DNA libraries were constructed using overlap extension PCR with end primers designed for Golden Gate assembly (templates and targeted positions for each round are summarized in table S1).^14^ Constructs were subcloned into an in-house made pET26-ccdB vector using *Bsa*I restriction enzyme.

**Shuffling by overlap extension PCR**

After each round of evolution, beneficial diversity was combined by DNA shuffling of fragments generated by overlap extension PCR. Primers were designed that encoded the identified mutation. These primers were used to generate short fragments (up to 4) which were gel-purified and mixed appropriately in overlap extension PCR to generate genes containing all possible combinations of mutations. Genes were cloned as described above.

**Library screening**

For protein expression and screening, all transfer and aliquoting steps were performed using a Hamilton liquid-handling robot. Chemically competent *E. coli* BL21 (DE3) were transformed with the appropriate library plasmid. Freshly transformed colonies were used to inoculate LB medium (140 *µ*L) supplemented with kanamycin (50 *µ*g mL^−1^) in Corning Costar 96-well microtiter round-bottom plates. Each plate also contained six freshly transformed clones of the parent template and two clones of pET26b_RFP as a negative control. Plates were incubated overnight (30 °C, 80% humidity, 850 r.p.m.), then an aliquot of overnight culture (20 *µ*L) was used to inoculate auto-inducible ZYM media (480 *µ*L) supplemented with kanamycin (50 *µ*g mL^−1^). Plates were incubated for 22 h (21 °C, 80% humidity, 850 r.p.m.). Cells were collected by centrifugation (2,900 × *g* for 10 min). The supernatant was discarded, and the pelleted cells were resuspended in a lysis mix consisting of BugBuster Protein Extraction reagent (50 *µ*L, 10 x concentrate diluted with Gly-OH (pH 9.7, 50 mM) to give a 1 x working solution) supplemented with DNase I (10 *µ*g mL^−1^). Cell lysis was initiated by incubation (30 °C, 30 min, 80% humidity, 850 r.p.m.) and the resulting lysate was diluted with Gly-OH reaction buffer (350 *µ*L, pH 9.7, 50 mM). Diluted lysates were subjected to a heat treatment (2 h, 65 °C, 850 r.p.m.) and cell debris was removed by centrifugation (2,900 × *g*, 10 min). 135–150 *µ*L of clarified cell lysate was transferred to a 96-deep-well plate containing reaction buffer (pH 9.7, 50 mM Gly-OH) and a single 3 mm *Gf*-PC disc in each well, to make a final reaction volume of 300 μL. Lysate volumes were optimized at the outset of each round of evolution. Plates were then foil-sealed and incubated for 2-24 h at 65 °C, after which reactions were terminated by the addition of 300 μL of a cold methanol containing 12.5 mM trifluoracetic acid. Following reaction quenching, plates were foil-sealed and incubated for 30 min (30 °C, 850 r.p.m.) and insoluble protein precipitate removed by centrifugation (2,900 × *g*, 10 min). 100 *µ*L of the resulting reaction supernatant was sampled for UPLC analysis in a fresh 96-well microtitre plate. The most active clones of each round were then subjected to a second screening round, where each clone was represented as a triplicate. All expression and screening protocols were as described above, apart from overnight culture preparation, where LB media was instead inoculated with 20 *µ*L of a glycerol stock of the original overnight cultures from the library screening round.

**PC film solvent-casting to prepare *SolC*-PC**

PC pellets (Makrolon 2407) were dissolved in DCM for 16 h (40 mg mL^-1^). 40 *µ*l PC solution was dispensed into the wells of a 96-well microplate (polypropylene (PP), flat bottom, chimney well). Plates were left in a fumehood (airflow rate ~0.5 m/s) for 16 h to allow solvent to fully evaporate.

**General procedure for analytical scale biotransformations**

Analytical scale biotransformations were performed on either a single 3 mm *Gf*-PC disc (punched from an amorphous commercial PC film of 0.25 mm thickness), or on a single amorphous *SolC*-PC solvent-cast film. Reactions were conducted in a 96-well microplate (polypropylene (PP), flat bottom, chimney well) using a 100 *µ*l reaction volume. Unless stated otherwise, reactions were conducted in 90 μL Gly-OH (50 mM, pH 9.7) supplemented with 4% (v/v) BugBuster, and were initiated by addition of 10 *µ*L of a 10 x enzyme stock of the relevant biocatalyst in storage buffer (pH 7.5, 50 mM Tris-HCl, 150 mM NaCl). Following incubation, reactions were quenched with 1 volume MeOH supplemented with TFA (1 M, 8% v/v) and shaken (850 r.p.m.) for 30 min. Precipitated protein and insoluble plastic debris was removed by centrifugation (2,900 x *g* for 20 min), and supernatants were transferred to a fresh plate containing MeOH (1:10 dilution) for UPLC analysis.

**Chromatographic analysis**

Analysis was carried out by UPLC on a 1290 Infinity II Agilent LC system with the DAD set to either 224 nm or 270 nm, using a Phenomenex Kinetex XB-C18 100 Å, 5 *µ*m, 50 × 2.1 mm, LC Column with a gradated solvent ratio method. Mobile phase A was water and mobile phase B was acetonitrile, both containing 0.1% trifluoracetic acid, with the flow rate fixed at 1.1 mL min^−1^ and an injection volume of 4 *µ*L. Following sample injection, the mobile phase was set to 30% mobile phase B, increased to 60% mobile phase B over 60 s, held at 60% B for 6 s, then stepped back down to 30 % mobile phase B over 6 s. Peaks were assigned by comparison to a standard curve prepared from commercial BPA.

For BHET hydrolysis reactions chromatographic analysis was carried out on the same system with the same column and the same buffers but with a gradient method of 5% acetonitrile to 95% acetonitrile over 5 minutes. Peaks were assigned by comparison to a standard curve made from bought chemical standards.

For BPA-dimer hydrolysis reactions chromatographic analysis was carried out on the same system with the same column and the same buffers but with a gradient method of 40% acetonitrile to 95% acetonitrile over 1 minute. Peaks were assigned by comparison to a standard curve made from bought BPA-OMe and characterized BPA-dimer.

**pH and buffer screen**

To investigate optimum pH for the reaction, analytical scale biotransformations were performed using *SolC*-PC in the stated buffer and pH with enzyme (5 *µ*M) at 65 °C, 850 r.p.m for 2hr. Reactions were quenched with 1 volume MeOH supplemented with TFA (1 M, 8% v/v), shaken (850 r.p.m.) for 30 min, centrifuged (2,900 x *g* for 20 min), diluted with MeOH (1:10), and analysed by UPLC using the above method (Figure S12).

**DMSO tolerance**

To investigate DMSO tolerance, analytical scale biotransformations were performed using *SolC*-PC incubated with enzyme (5 *µ*M) in pH 9.7 Gly-OH (50 mM) with either 5%, 10%, 15%, 20%, 25%, 30%, 35% and 40% (v/v) DMSO cosolvent. All reactions were incubated (65 °C) and shaken (850 r.p.m.) in an Infors-HT plate shaking incubator. Reaction time course data was established using an individual microtiter plate containing the range of conditions tested for each time point. Reactions were quenched with 1 volume MeOH supplemented with TFA (1 M, 8% v/v), shaken (850 r.p.m.) for 30 min, centrifuged (2,900 x *g* for 20 min), diluted with MeOH (1:10), and analysed by UPLC using the above method.

**Temperature-activity profile assays**

To evaluate the activity of the LCC^ICCG^, PC-1, and PC-2 at elevated temperatures, biotransformations were performed using *SolC*-PC incubated with enzyme (1 *µ*M, 5 *µ*M, 10 *µ*M) in pH 9.7 Gly-OH (50 mM) with 40% DMSO cosolvent (v/v). All reactions were incubated at the stated temperature (65-80 °C) and shaken (850 r.p.m.) in an Eppendorf Thermomixer C tabletop plate shaker. Reaction time course data was established using an individual microtiter plate containing the range of conditions tested for each time point. Reactions were quenched with 1 volume MeOH supplemented with TFA (1 M, 8% v/v), shaken (850 r.p.m.) for 30 min, centrifuged (2,900 x *g* for 20 min), diluted with MeOH (1:10), and analyzed by UPLC. Elevated temperature screening was conducted in an Eppendorf Thermomixer C tabletop plate shaker due to a 65 °C operational limit of the Infors-HT plate shaking incubator used for evolution and previous assays; a slight difference was noted in the reaction profile of assay conducted under the same conditions in these differing apparatus (Figure S13).

**Protein melting temperature (*T* _m_) analysis**

The melting temperatures (*T*_m_) of PC-2 and LCC^ICCG^ were determined using differential scanning fluorimetry. For each protein, a 50 μl sample of 5 μM protein was prepared in buffer (pH 9.7, 50 mM Gly-OH) with a final concentration of 10X SYPRO Orange dye stock solution (Sigma-Aldrich) in an optically clear, lidded PCR tube (Bio-rad). Differential scanning fluorimetry melt-curve experiments were conducted using a Bio-rad CFX Connect 96 Real-Time PCR system set on the fluorescence resonance energy transfer channel to use the 450/490 excitation and 560/580 emission filters. The temperature was increased from 25 to 95 °C with an increment of 0.3 °C s^−1^. For both PC-2 and LCC^ICCG^ the melting temperatures were measured to be >95 °C. 95 °C is the maximum temperature of the instrument.

**SEM analysis of enzymatically degraded PC**

To prepare degraded PC samples for SEM imaging, analytical scale biotransformations were performed using both *Gf*-PC and *SolC*-PC incubated with enzyme (5 *µ*M) in pH 9.7 Gly-OH (50 mM) supplemented with either 4% BugBuster or 40% DMSO (v/v). Reactions were incubated (65 °C) and shaken (850 r.p.m.) in an Infors-HT plate shaking incubator. PC substrate was then removed and rinsed twice with DI water to remove soluble enzyme and buffer components, and once with EtOH to aid with drying.

Samples were analysed by SEM as follows: polymer samples were sputter coated with Au/Pd (thickness 5 nm) to ensure conductivity and prevent charging during SEM imaging. SEM imaging was performed using the in-lens detectors in a Zeiss Ultra 55 Quanta 250 FEG-SEM with an accelerating voltage of 5 kV and probe current of approximately 2 nA.

**Characterisation of PC substrates**

The number and weight average molecular weights (M_n_ and M_w_) of polymer chains were determined by GPC. PC samples (10 mg) were dissolved in HPLC-grade chloroform (2 mL) for 16 h with stirring. The solution was filtered through a 0.24 *µ*m PTFE filter and GPC analysis was conducted using an Agilent 1260 Infinity II Multi-Detector Gel Permeation Chromatography system equipped with RUI and diode-array detectors. Columns were packed with PL-gel 10 *µ*m MIXED-B beads. The mobile phase used was HPLC-grade chloroform at a flow rate of 1 mL min^-1^ (35 °C).

Polymer crystallinity was determined using DSC, using between 4 - 6 mg of material. DSC data were obtained using a DSC 2500 TA instrument. Samples were run in triplicate, in series, over a −50 to 300°C temperature range under a nitrogen atmosphere at a heating rate of ±10°C min^−1^ in a sealed Tzero aluminium crucible.

**Analytical scale biotransformation with bis(2-hydroxyethyl) terephthalate (BHET)**

Biotransformations were performed using either PC-2 or LCC^ICCG^ (0.05 *µ*M) in Gly-OH (50 mM, pH 8.7) supplemented with 40% DMSO cosolvent. A total reaction volume of 1 mL containing 2 mM BHET was incubated at 65 °C, 850 r.p.m.. Conversion of BHET was monitored through periodical sample of the reaction mixture (50 *µ*L). Samples were immediately quenched with 1 volume MeOH supplemented with TFA (1 M, 8% v/v) and submitted for UPLC analysis.

**Analytical scale biotransformation with BPA-dimer**

Biotransformations were performed using either PC-2 or LCC^ICCG^ (0.05 *µ*M) in Gly-OH (50 mM, pH 8.7) supplemented with 40% DMSO cosolvent. A total reaction volume of 1 mL containing 0.5 mM BPA-dimer was incubated at 40 °C, 850 r.p.m.. Conversion of the BPA-dimer was monitored through periodical sample of the reaction mixture (50 *µ*L). Samples were immediately quenched with 1 volume MeOH supplemented with TFA (1 M, 8% v/v) and submitted for UPLC analysis.

**Molecular dynamics simulations and docking**

MD simulations of LCC^ICCG^ (PDB: 6THT)^9^ and an AlphaFold2 model of PC-2 were carried out using Gromacs 2024.2^16,17^ with the Amber ff14SB force field^18^ with a solvation box with a minimum 10 Å buffering distance around the protein and counter-ions generated using AmberTools. Simulations were performed using constant temperature (velocity-rescaling thermostat,^19^ 300 K) and pressure (Parrinello-Rahman barostat,^20^ 1 bar), 10 Å van der Waals and electrostatic cutoffs, particle mesh Ewald for long-range electrostatics, LINCS bond constraints on bonds involving hydrogen,^21^ periodic boundary conditions and a 2 fs timestep. After energy minimisation, two sets of MD simulations were run for each enzyme variant using the following protocol: (i) 1 ns constant volume (NVT) equilibration of the solvent with 10 kJ mol^-1^ Å^-2^ positional constraints on the protein non-hydrogen atoms; (ii) 1 ns constant pressure (NPT) equilibration with the same positional constraints; (iii) 500 ns of unconstrained production MD.

Following MD, 4D docking (ICM Pro)^22^ of a BPA dimer was performed against a series of frames along the trajectory resulting in an RTCNN score of -18.44. In the resultant docked pose, the BPA dimer occupies the binding cleft in a catalytically competent orientation with a contact footprint of 567Å^2^.

**Chemical synthesis of bis(4-(2-(4-methoxyphenyl)propan-2-yl)phenyl) carbonate (BPA-dimer)**

To a solution of 4-[1-(4-methoxyphenyl)-1-methylethyl]phenol (800 mg, 3.301 mmol, 4 equiv) and 1,1'-carbonyldiimidazole (134 mg, 0.825 mmol, 1 equiv) in acetonitrile (20 mL) was added DMAP (10.1 mg, 0.0825 mmol, 0.1 equiv.), and the resulting mixture was stirred at 80 °C for 16 h. The reaction mixture was concentrated *in vacuo,* and the residue was purified by flash column chromatography (SiO_2_, hexane/EtOAc: 7/1) to afford the title compound as a colourless solid (95 mg, 22%). ^1^H NMR (400 MHz, CDCl_3_) δ 7.31 – 7.26 (m, 4H), 7.22 – 7.13 (m, 8H), 6.89 – 6.79 (m, 4H), 3.80 (s, 6H), 1.69 (s, 12H).

# **Supplementary Figures**

**
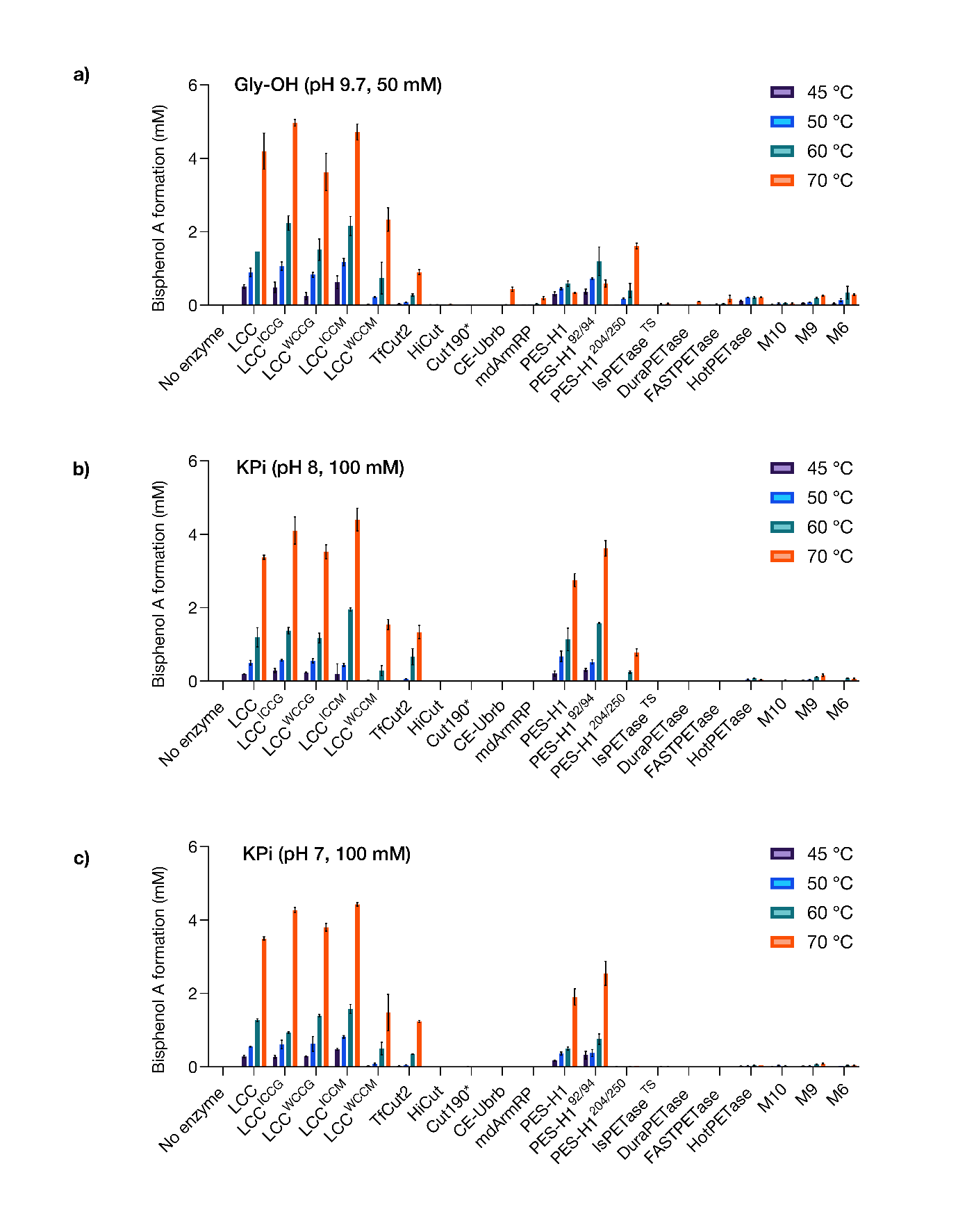
**

**Figure S1:** Bar chart showing the mean total concentration of BPA produced by a panel of hydrolases following incubation with a *Gf*-PC disc in (**a**) pH 9.7 Gly-OH buffer, (**b**) pH 8 KP_i_ buffer, or (**c**) pH 7 KPi buffer. Biotransformations were performed as 100 *µ*L reactions with a single *Gf*-PC disc, enzyme (0.5 *µ*M), and supplemented with 4% (v/v) BugBuster, 20 h incubation at 45–70 °C, 850 r.p.m.. Error bars represent the standard deviation of measurements made in triplicate.

**
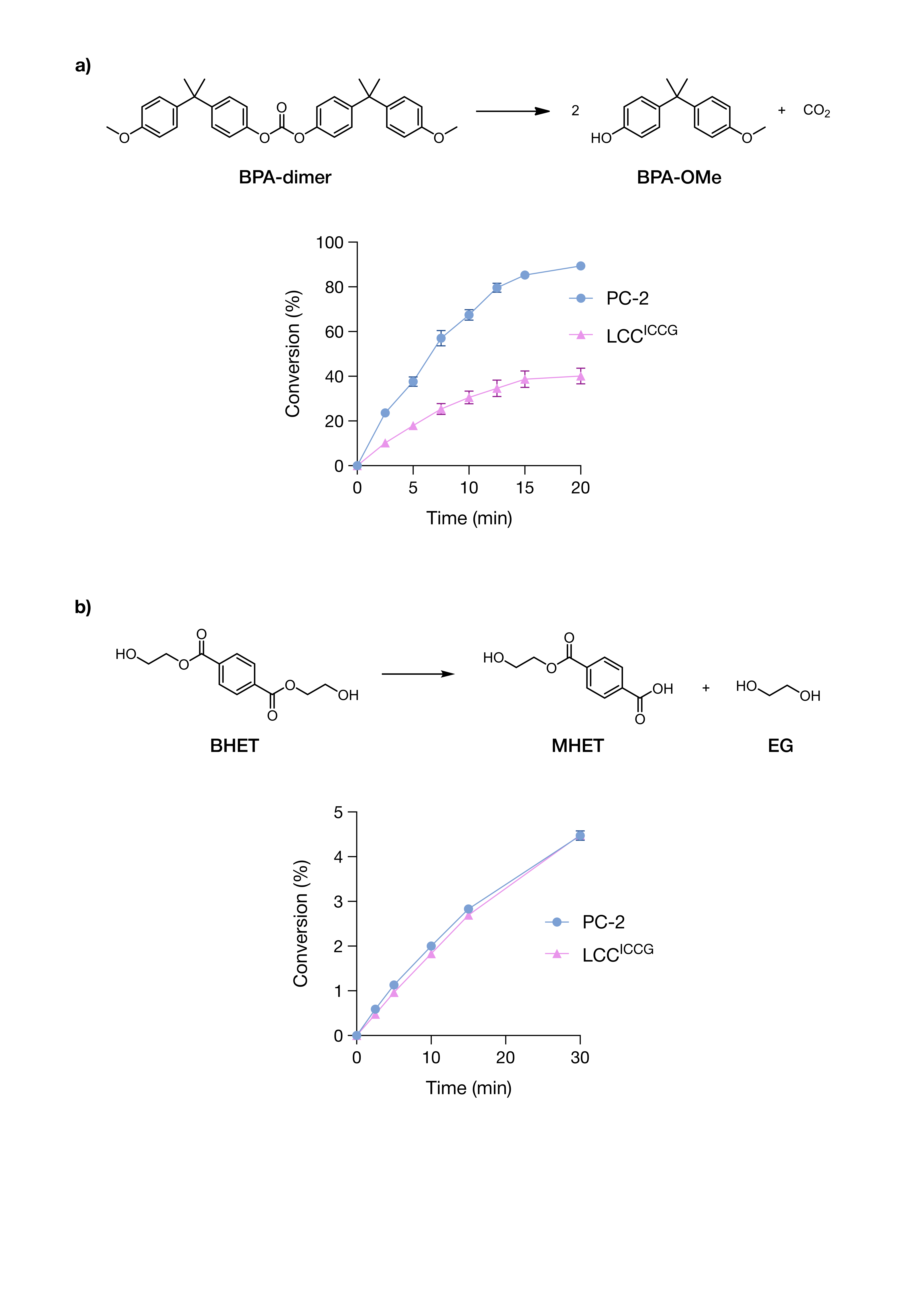
**

**Figure S2:** Hydrolysis of model small molecule substrates by PC-2 and LCC^ICCG^. (**a**) Hydrolysis of a chemically synthesized BPA-dimer. Biotransformations were performed as 1 mL reactions with 0.5 mM BPA-dimer, enzyme (0.05 *µ*M), Gly-OH (50 mM, pH 9.7), 40 °C, 850 r.p.m.. Error bars represent the standard deviation of measurements made in triplicate. (**b**) Hydrolysis of BHET. Biotransformations were performed as 1 mL reactions with 2 mM BHET, enzyme (0.05 *µ*M), Gly-OH (50 mM, pH 9.7), 65 °C, 850 r.p.m.. Error bars represent the standard deviation of measurements made in triplicate.

**
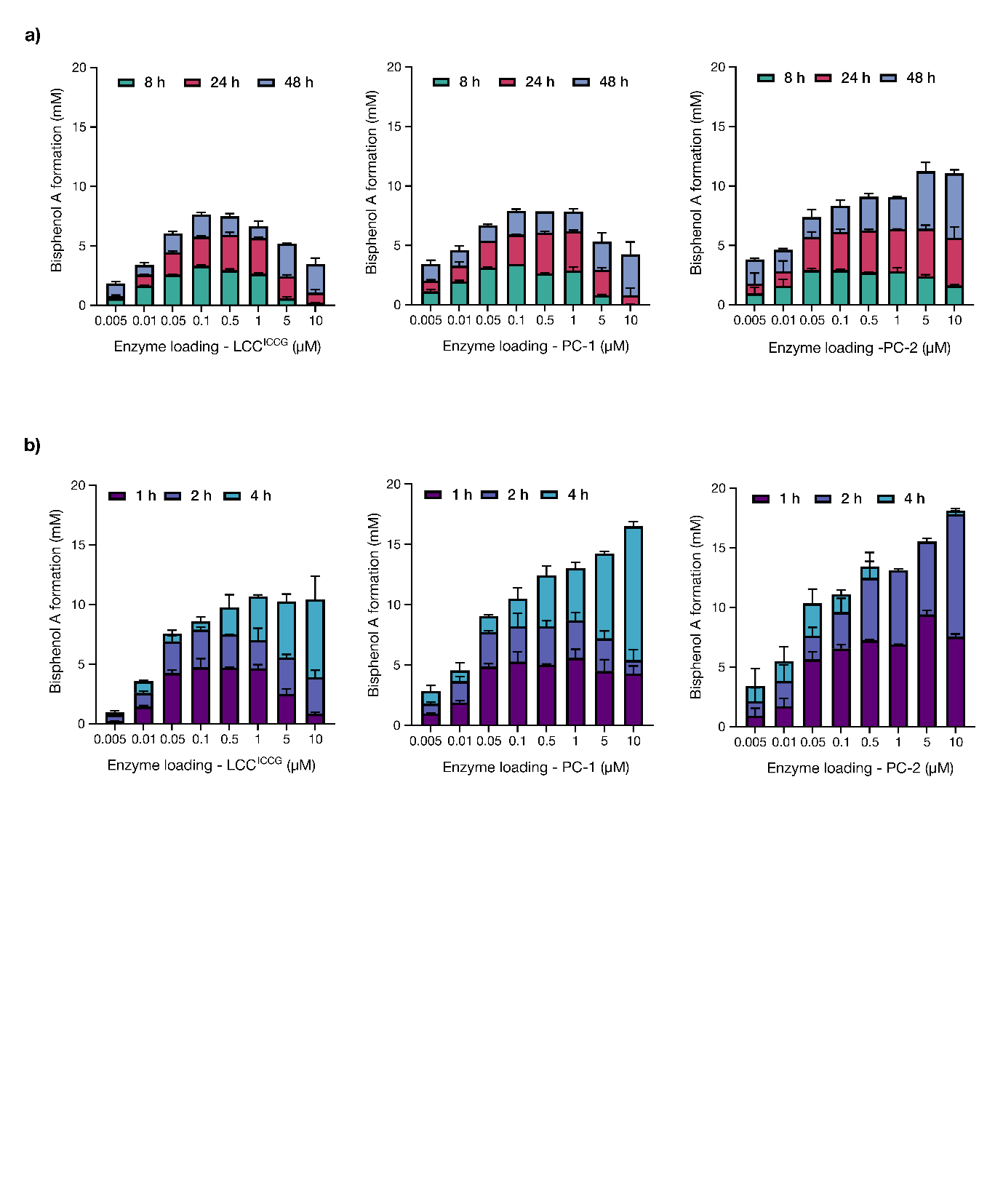
**

**Figure S3:** Cumulative bar charts showing the mean total concentration of BPA produced by LCC^ICCG^ (left), PC-1 (centre), and PC-2 (right), over a range of enzyme concentrations using either a *Gf*-PC disc (**a**) or *SolC*-PC film (**b**). Biotransformations were performed as 100 *µ*L reactions with either *Gf*-PC disc or *SolC*-PC film, enzyme (variable concentration), Gly-OH (50 mM, pH 9.7) supplemented with 4% (v/v) BugBuster, 65 °C, 850 r.p.m.. Error bars represent the standard deviation of measurements made in triplicate.

**
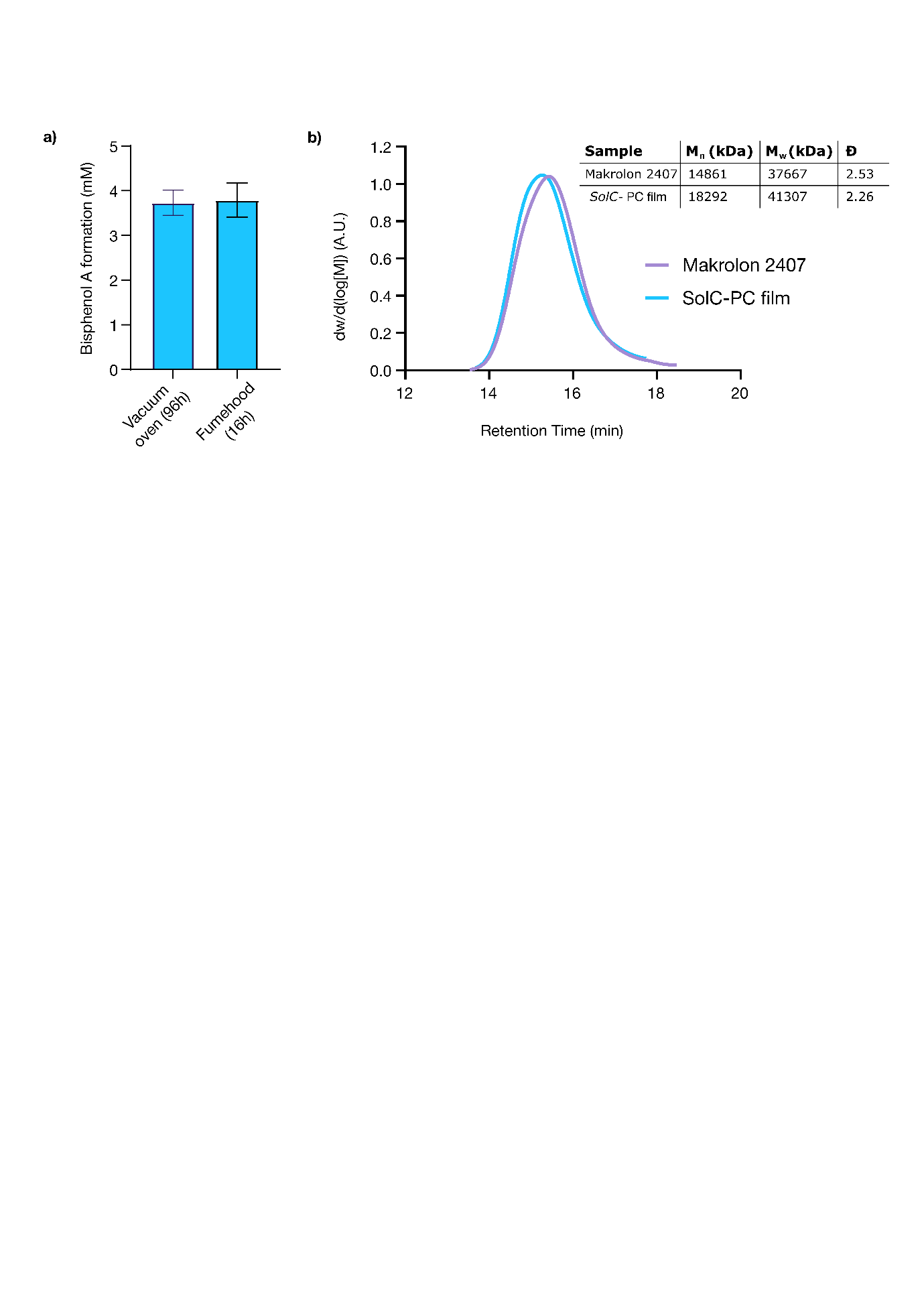
**

**Figure S4: (a)** Bar chart showing the mean total concentration of BPA produced by PC-1 (5 *µ*M) from *SolC*-PC films dried in different ways to investigate the effect of residual solvent on enzyme activity. Biotransformations were performed as 100 *µ*L reactions with *SolC*-PC films, PC-1 (5 *µ*M), Gly-OH (50 mM, pH 9.7) supplemented with 4% (v/v) BugBuster, 1 h, 65 °C, 850 r.p.m.. (**b**) GPC chromatogram (CHCl_3_) of PC samples before and after the solvent casting process (dried in a fumehood (airflow rate ~0.5 m/s) for 16 h) validating that shear from pipetting of solution-phase PC had not cause significant chain length reduction. GPC samples were calibrated against polystyrene standards.

**
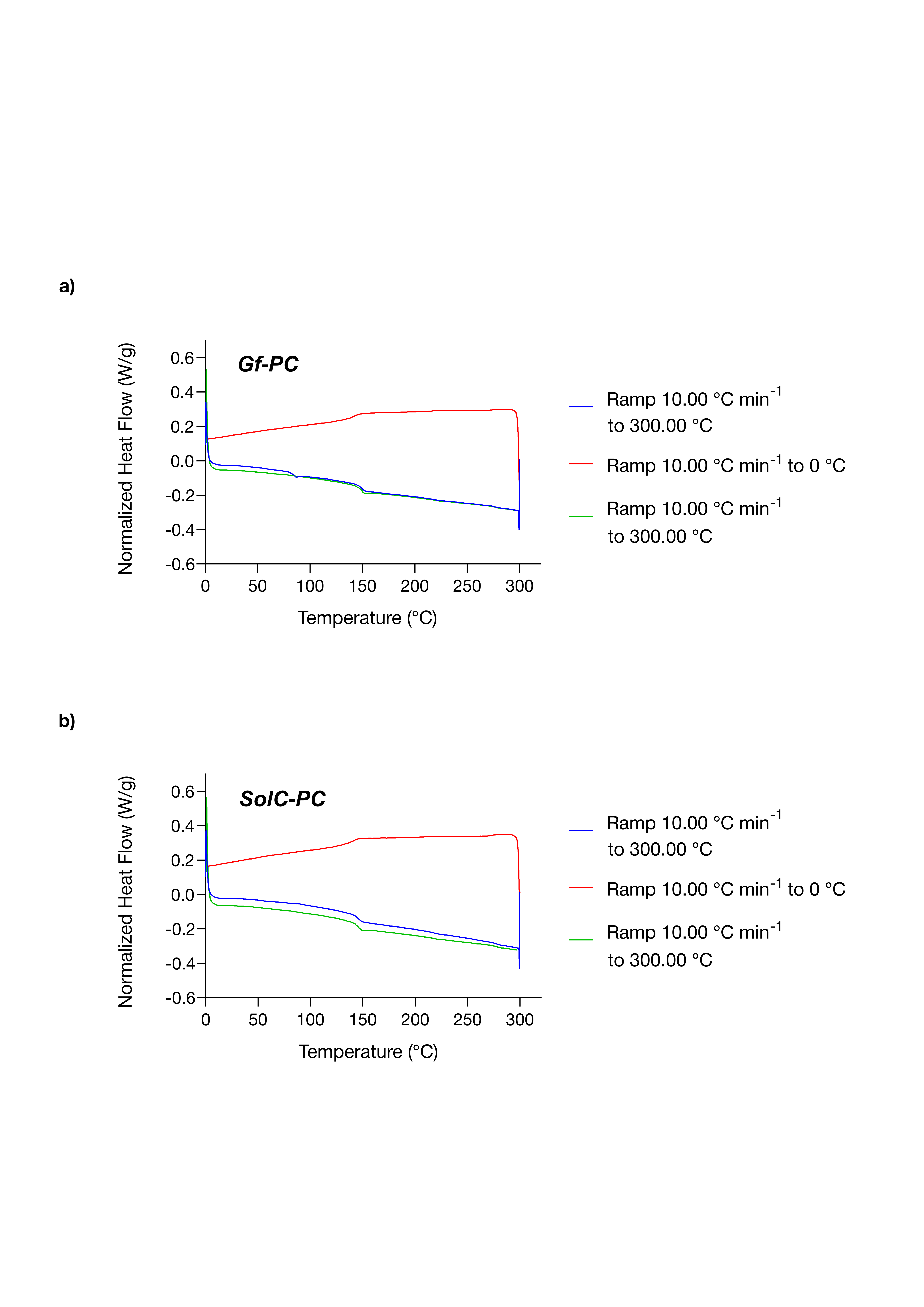
**

**Figure S5:** **DSC thermogram of commercial *Gf*-PC film and solvent cast *SolC*-PC film.** Both films were determined to be amorphous based the absence of a significant endotherm at the *T_g_* (~149.5 °C) observed on the first temperature ramp (blue).

**
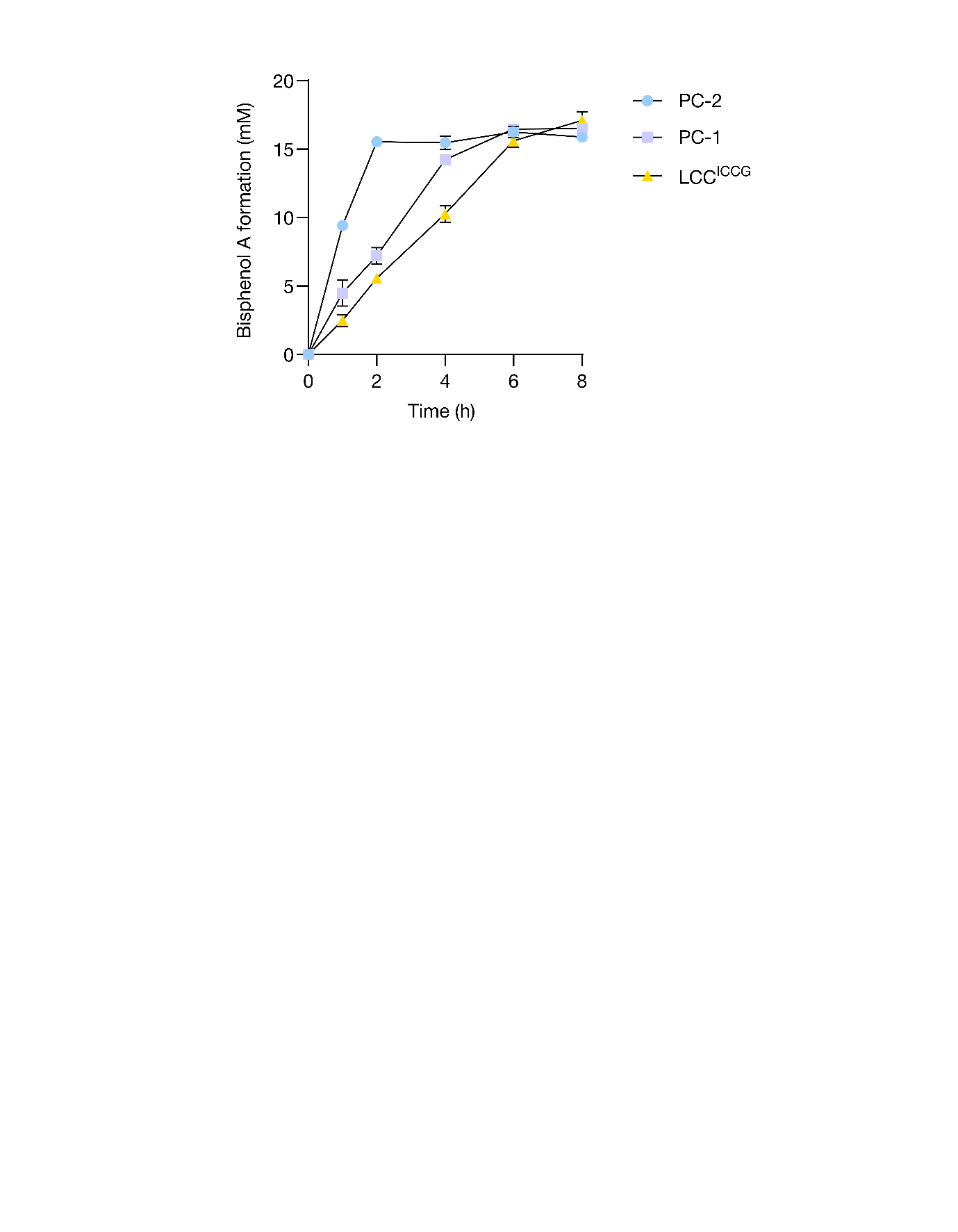
**

**Figure S6:** Time-course of the concentration of BPA produced by PC-2, PC-1, and LCC^ICCG^ over 8 h, showing the reaction stalling at ~15.5 mM BPA formation. Biotransformations were performed as 100 *µ*L reactions with a single *SolC*-PC film, enzyme (5 *µ*M), Gly-OH (pH 9.7, 50 mM) supplemented with 4% (v/v) BugBuster, 65 °C, 850 r.p.m.. Error bars represent the standard deviation of measurements made in triplicate.

**
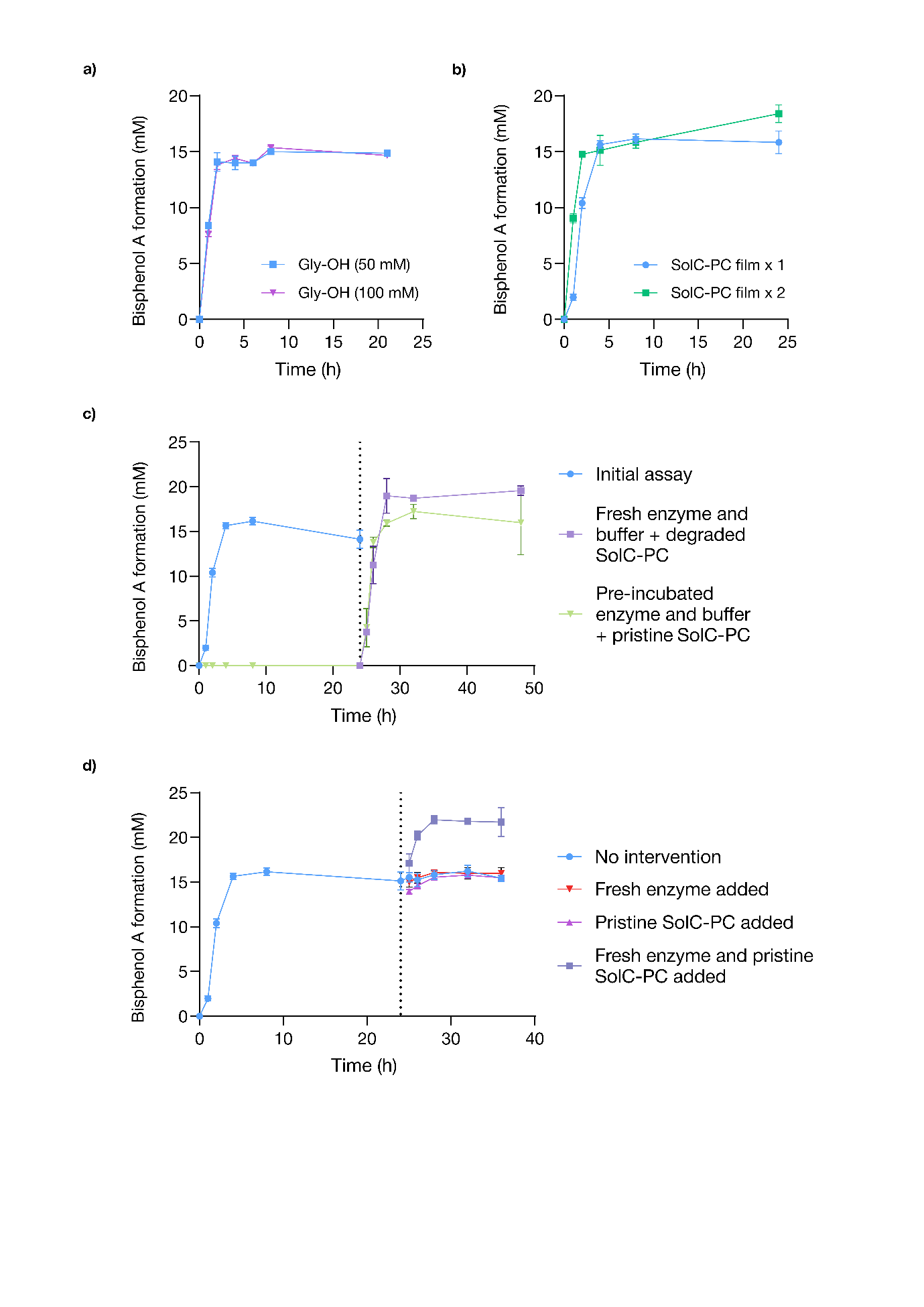
**

**Figure S7:** (**a**) 24 h time-course of the concentration of BPA produced by PC-2 (5 *µ*M) in either 50 mM or 100 mM Gly-OH (pH 9.7). Biotransformations were performed as 100 *µ*L reactions with a single *SolC*-PC film, PC-2 (5 *µ*M), Gly-OH (pH 9.7) supplemented with 4% (v/v) BugBuster, 65 °C, 850 r.p.m. (**b**) 24 h time-course of the concentration of BPA produced by PC-2 (5 *µ*M) from *SolC*-PC film (either one film or two films). (**c**) 24 h time-course of the concentration of BPA produced by PC-2 (5 *µ*M) from a single *SolC*-PC film (blue). After 24 h the partially degraded substrate from this assay was added to fresh enzyme and buffer and incubated for a further 24 h (purple). Additionally, PC-2 (5 *µ*M) was incubated in buffer at 65 °C for 24 h, at which point a pristine *SolC*-PC film was added and incubation was continued for a further 24 h (green). (**d**) 24 h time-course of the concentration of BPA produced by PC-2 (5 *µ*M) from *SolC*-PC film, at which point reaction interventions were performed as per the figure legend. Biotransformations for **b**, **c** and **d** were performed as 100 *µ*L reactions with a single *SolC*-PC film, PC-2 (5 *µ*M), Gly-OH (pH 9.7, 50 mM) supplemented with 4% (v/v) BugBuster, 65 °C, 850 r.p.m. Error bars represent the standard deviation of measurements made in triplicate.

**
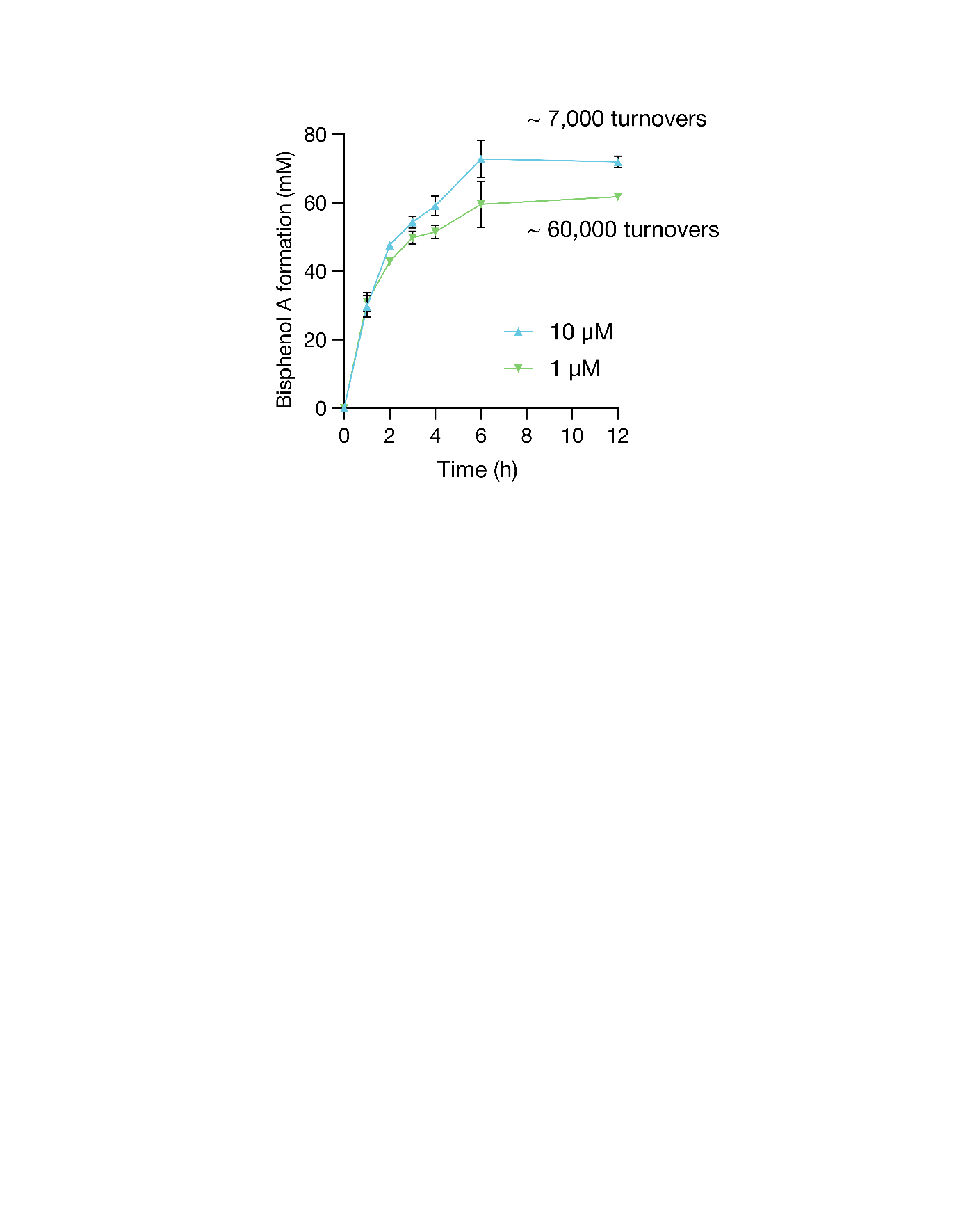
**

**Figure S8:** Time-course of the concentration of BPA produced by PC-2 at an enzyme loading of either 1 *µ*M (green) or 10 *µ*M (blue). Biotransformations were performed as 100 *µ*L reactions with *SolC*-PC film, enzyme (10 *µ*M), Gly-OH (50 mM, pH 9.7) supplemented with 40% (v/v) DMSO, 850 r.p.m.. Error bars represent the standard deviation of measurements made in triplicate.

**
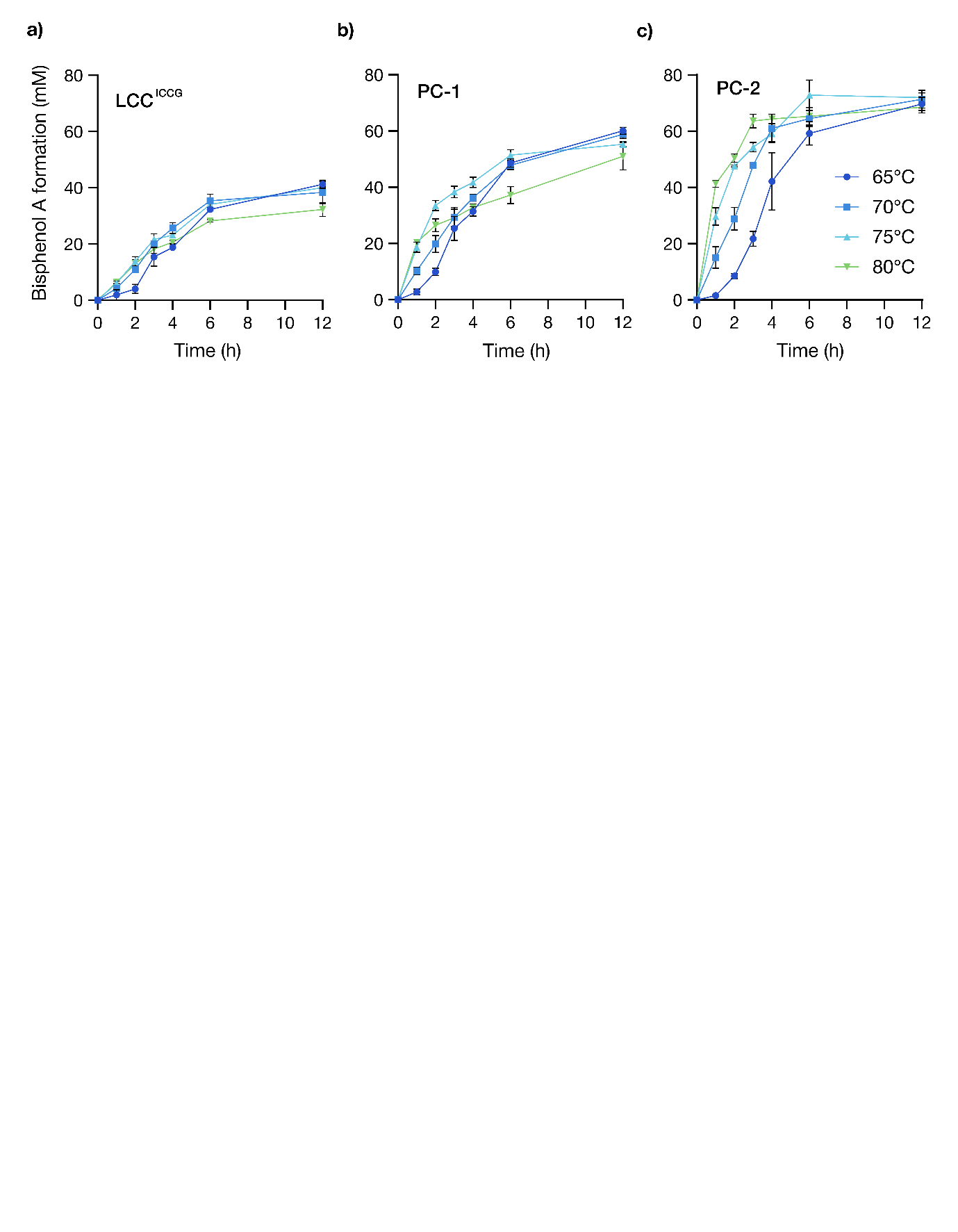
**

**Figure S9:** Time-courses of the concentration of BPA produced by LCC^ICCG^ (**a**), PC-1 (**b**), and PC-2 (**c**) following incubation at 65 °C, 70 °C, 75 °C and 80 °C. Biotransformations were performed as 100 *µ*L reactions with *SolC*-PC film, enzyme (10 *µ*M), Gly-OH (50 mM, pH 9.7) supplemented with 40% (v/v) DMSO, 850 r.p.m.. Error bars represent the standard deviation of measurements made in triplicate.

**
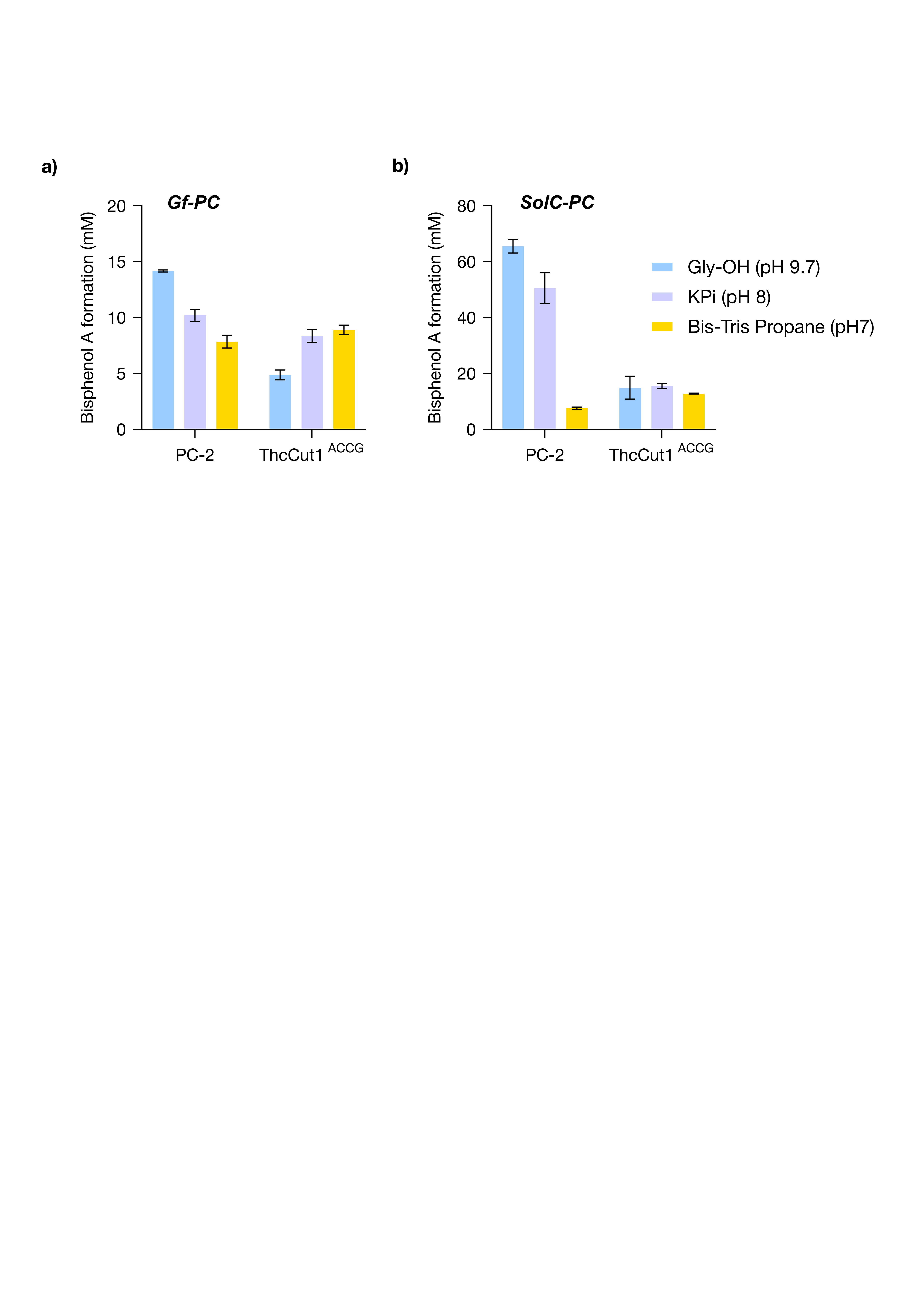
**

**Figure S10:** Comparison of BPA monomer release catalyzed by PC-2 and ThcCut1^ACCG^ (**a**) BPA release from biotransformations performed on commercial *Gf*-PC film following 12 h incubation, and (**b**) solvent cast *SolC*-PC film following 4 h incubation. General reaction conditions: enzyme (5 *µ*M), 65 °C, 850 r.p.m. various buffers supplemented with 40 % DMSO cosolvent. Error bars represent the standard deviation of measurements made in duplicate.

**

**

**Figure S11:** Comparison of MD simulations of LCC^ICCG^ (blue, PDB:6THT^9^) and an Alphafold2 model of PC-2 (orange). Distributions for the 125_cα_126_cα_-127_cα_128_cα_ dihedral angle (left) and distance between loop A and B (right).

**
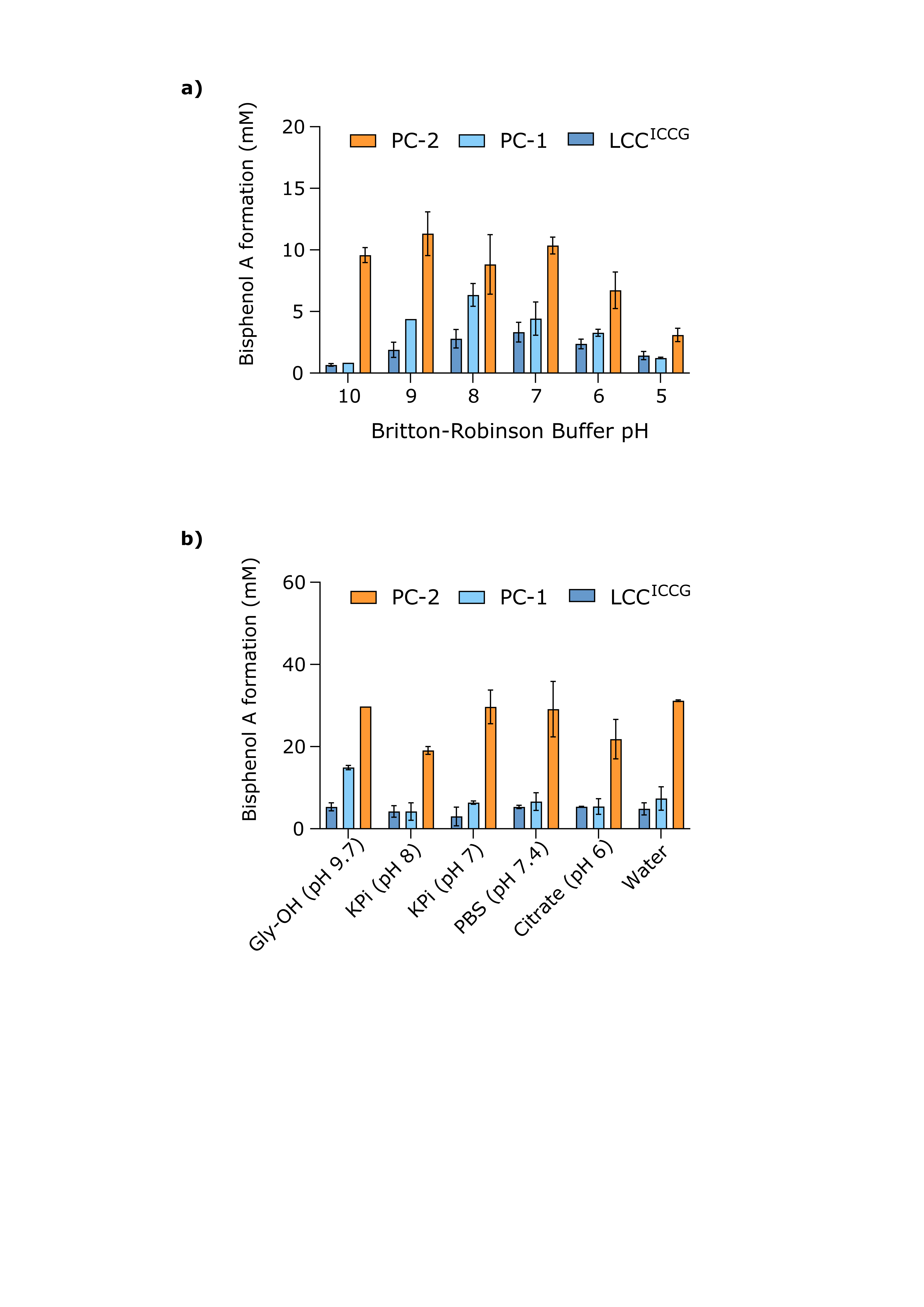
**

**Figure S12:** pH screen of evolved PC-2 compared to its evolutionary precursors. (**a**) Bar chart of BPA formation catalyzed by LCC^ICCG^, PC-1, and PC-2 (5 *µ*M) after 2 h incubation in Britton-Robinson (“universal”) buffer using a pH range of pH 5 – 10. Reaction conditions: *SolC*-PC film, enzyme (5 *µ*M), 100 *µ*L total reaction volume, 65 °C, 850 r.p.m. Error bars represent the standard deviation of measurements made in triplicate. (**b**) Bar chart of BPA formation catalyzed by LCC^ICCG^, PC-1, and PC-2 (5 *µ*M) after 2 h incubation in a range of common buffers supplemented with 40% DMSO. Reaction conditions: single *SolC*-PC film, enzyme (5 *µ*M), 100 *µ*L total reaction volume, 65 °C, 850 r.p.m.. Error bars represent the standard deviation of measurements made in triplicate.

**
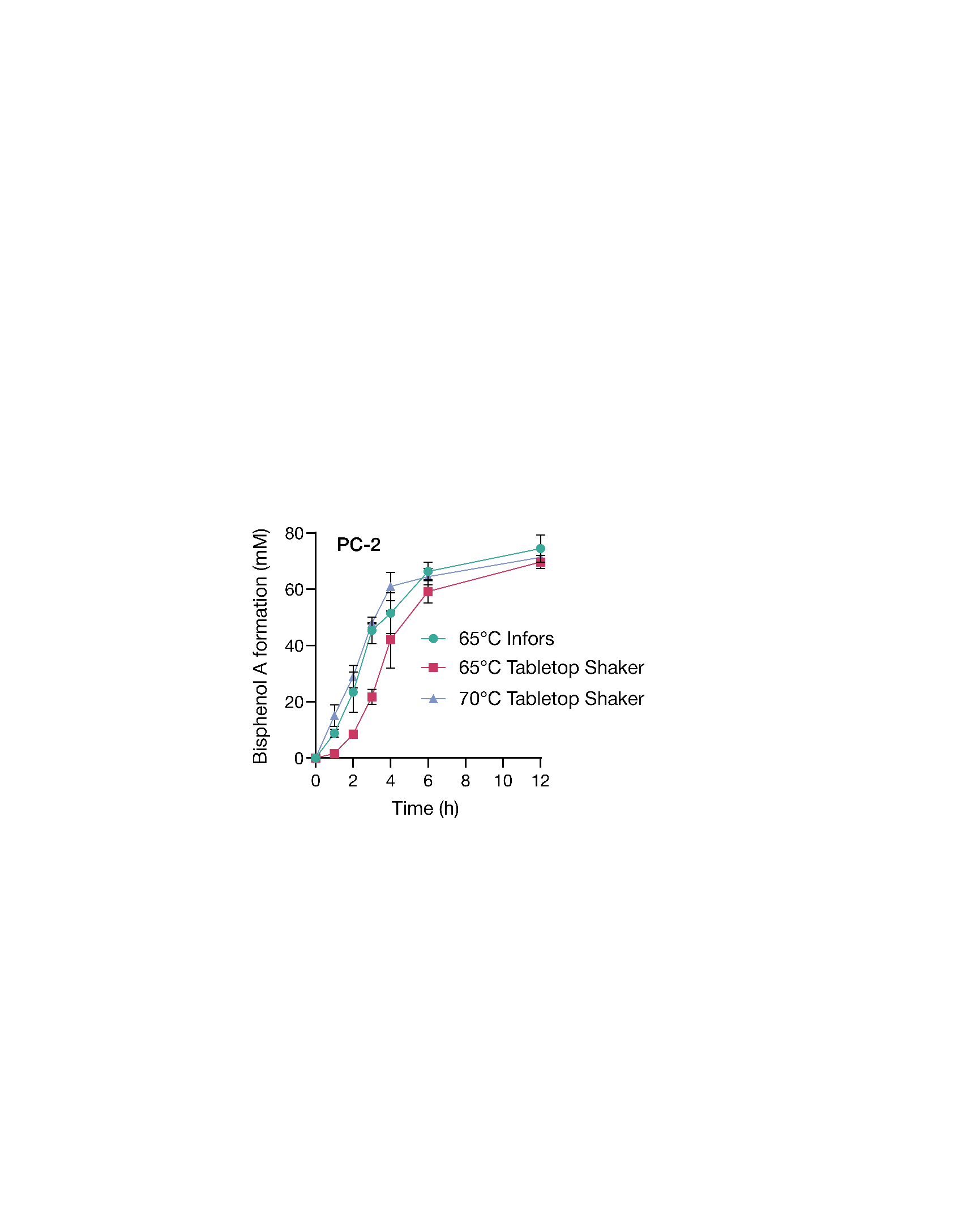
**

**Figure S13:** Comparison of differing concentrations of BPA produce by PC-2 with reactions conducted using either a Infors-HT plate shaking incubator or an Eppendorf Thermomixer C tabletop plate shaker. It was noted that comparable reaction profiles were obtained from time-course reactions conducted in the Infors-HT at 65 °C and the Eppendorf Thermomixer C at 70 °C, with slower monomer formation observed in the Eppendorf Thermomixer C at 65 °C. Biotransformations were performed as 100 *µ*L reactions with *SolC*-PC film, PC-2 (10 *µ*M), Gly-OH (50 mM, pH 9.7) supplemented with 40% (v/v) DMSO, 850 r.p.m.. Error bars represent the standard deviation of measurements made in triplicate.


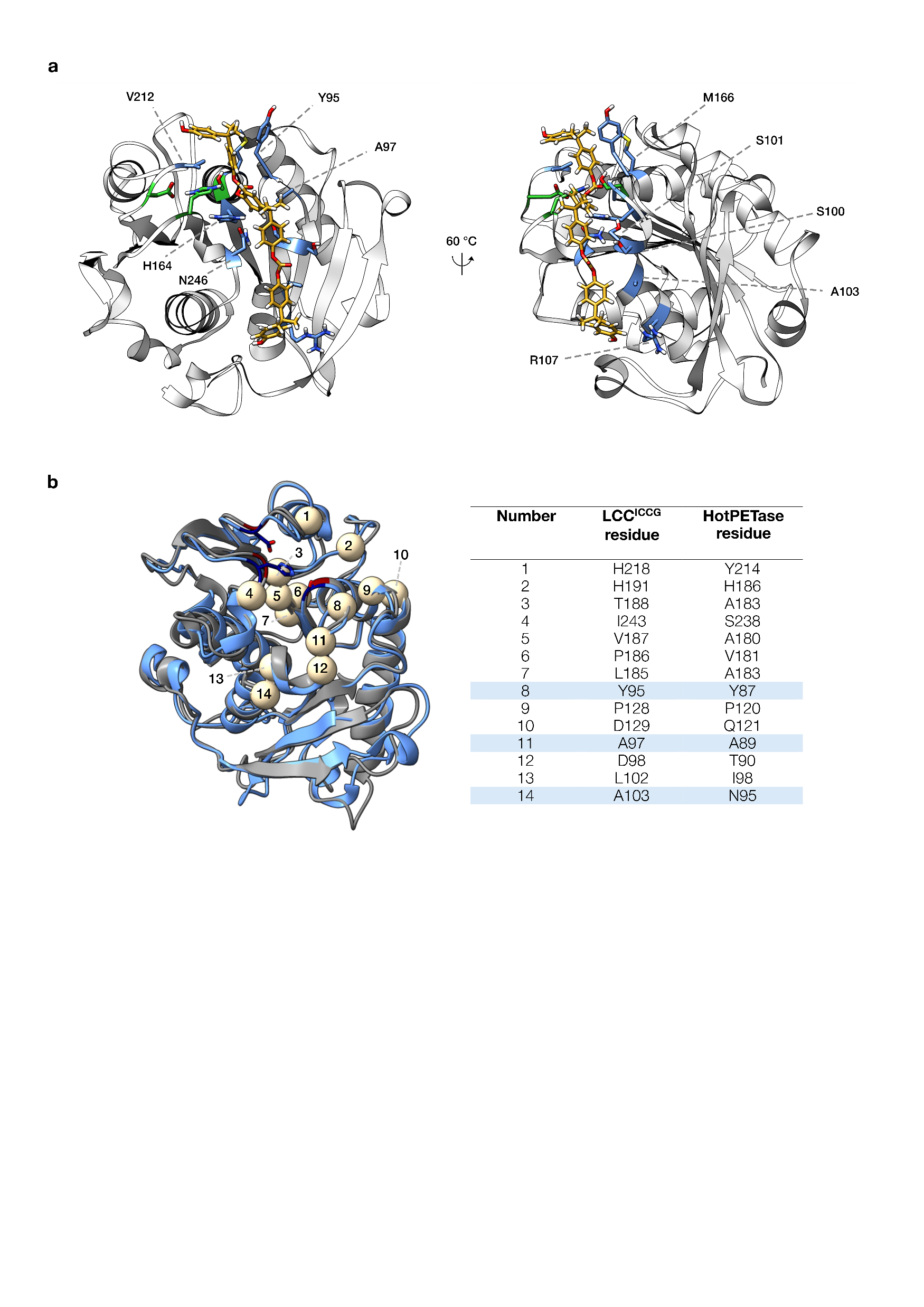

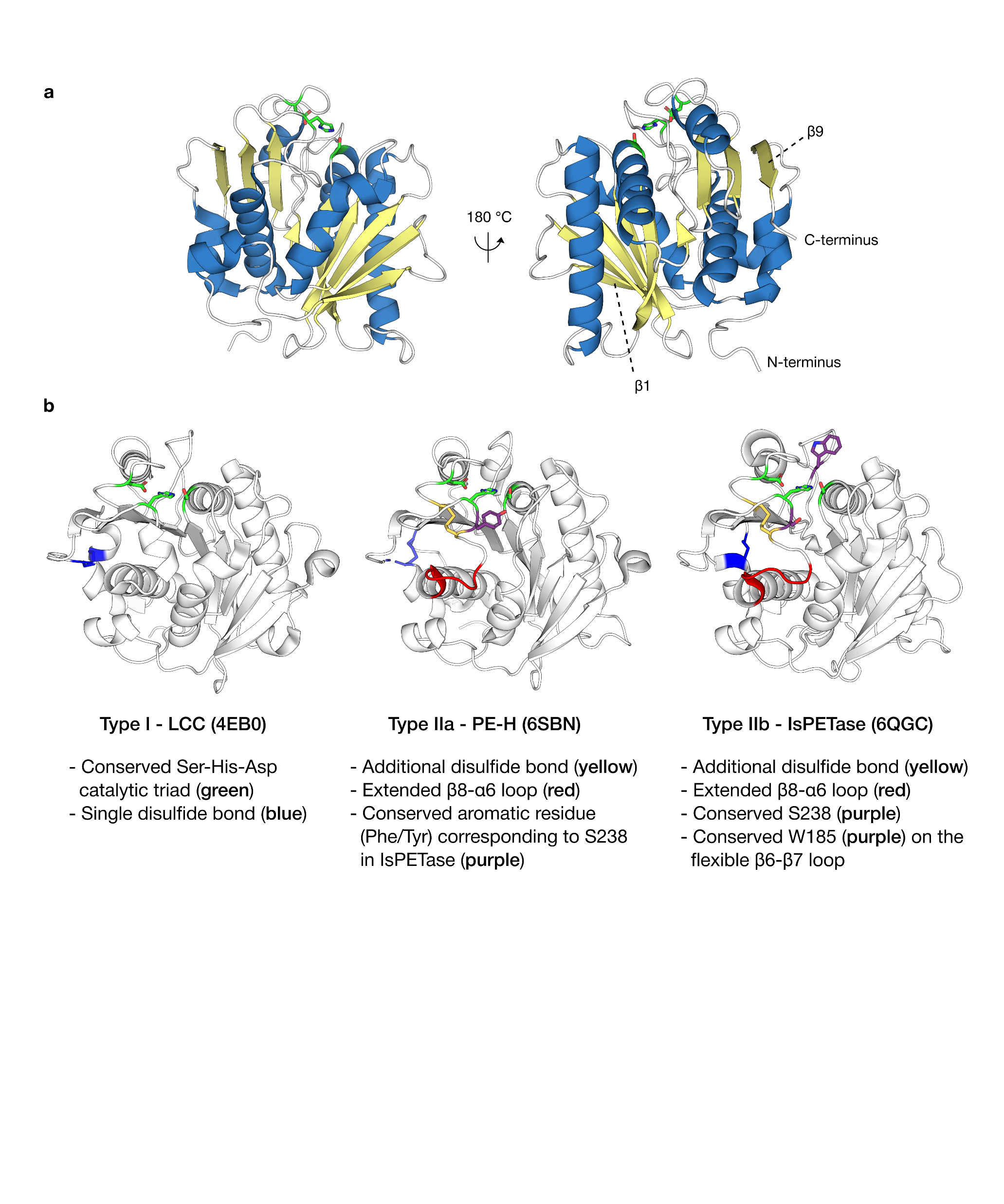


**Figure S14:** Docking of PC-trimer into LCC^ICCG^ (PDB: 6THT)^9^ to guide selection of positions for site saturation for round 1 of evolution. The PC-trimer was modelled in Avogadro using the smiles string below. This PC-trimer was docked into LCC^ICCG^ using AutoDock Vina 4. The lowest energy pose of the docked PC-trimer is shown in stick representation as yellow carbons with the catalytic triad of LCC^ICCG^ shown as green atom-coloured sticks. Residues within a 3 Å distance of the PC-trimer were selected for mutagenesis and are shown in stick representation with blue carbons.

Smiles string:

(CC(C)(c1ccc(O)cc1)c6ccc(OC(=O)Oc5ccc(C(C)(C)c4ccc(OC(=O)Oc3ccc(C(C)(C)c2 ccc(O)cc2)cc3)cc4)cc5)cc6).

**
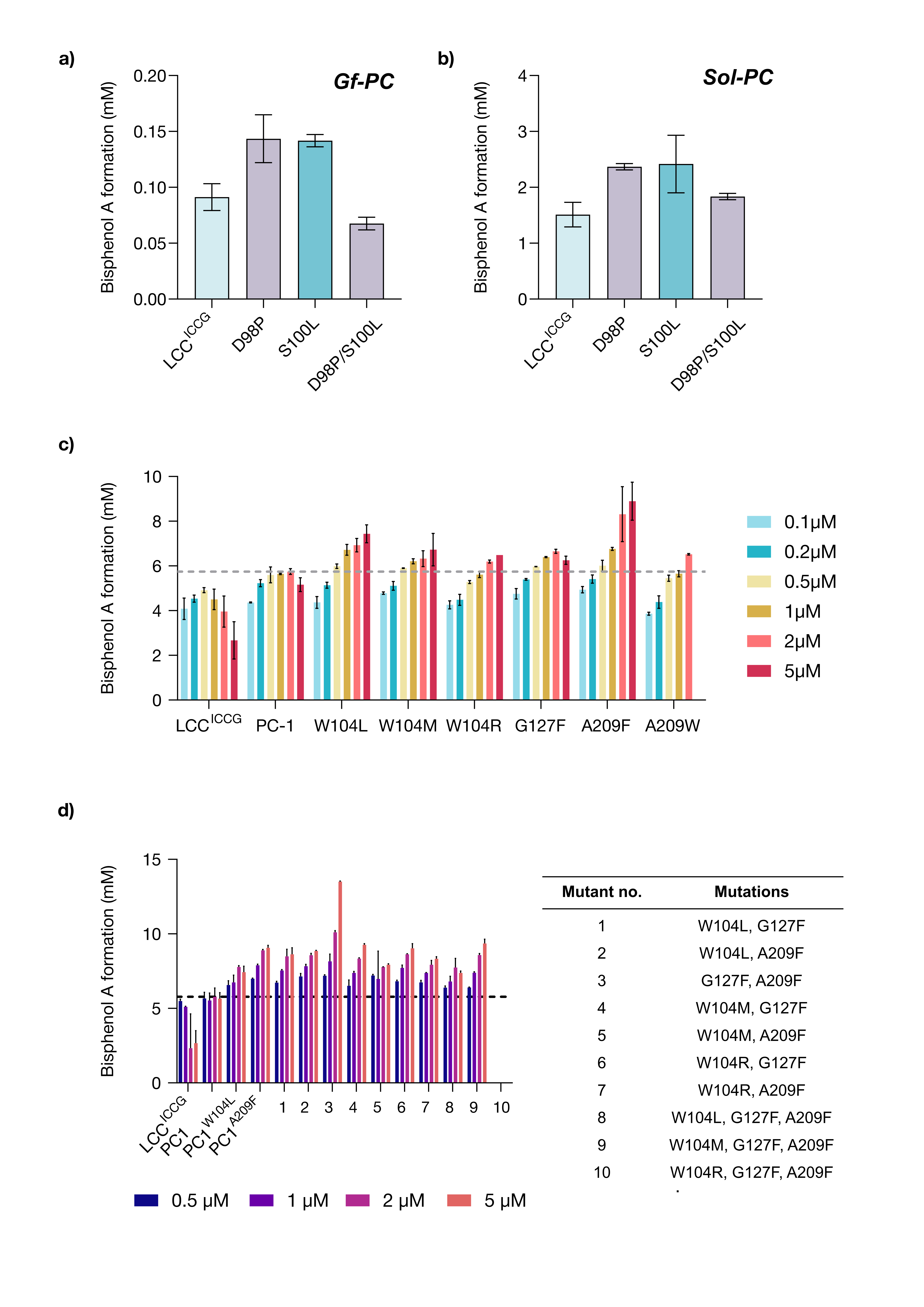
**

**Figure S15:** Summary of activity data used to select improved PC hydrolase variants throughout evolution. **(a-b)** BPA release catalysed by purified mutants of LCC^ICCG^ identified in the first round of evolution. Reaction conditions: either *Gf*-PC discs (**a**) or *SolC*-PC film (**b**), 0.5 *µ*M enzyme, Gly-OH (50 mM, pH 9.7) supplemented with 4 % BugBuster, 3 mL total reaction volume, 8 h, 65 °C, 200 r.p.m.. (**c**) Concentration-activity profiling of purified single point mutants identified from the second round of evolution lysate screening, and (**d**) Concentration-activity profiling of the subsequent shuffled variants. Variant 3 was selected as the best hits and named PC-2. Reaction conditions: *SolC*-PC film, enzyme (variable concentration), Gly-OH (50 mM, pH 9.7) supplemented with 4% BugBuster, 100 *µ*L total reaction volume, 2 h, 65 °C, 850 r.p.m. Error bars represent the standard deviation of measurements made in duplicate.

# **Supplementary Tables**

**Table S1:** Directed evolution strategy and results. Residues mutated and reasons for their selection for each directed evolution round are detailed. Beneficial mutations are also detailed.

| **Round** | **Description** | **Screening conditions** | **Beneficial mutations** | **Best variant** |
| --- | --- | --- | --- | --- |
| 1 | Saturation mutagenesis of positions identified by computational docking of a model PC trimer into LCC^ICCG^ (Figure S14): Y95, A97, S100, S101, A103, R107, H164, M166, V212, N246.  Structural overlay of positive mutations from the evolution of HotPETase^9,2^: D98, L102, P128, D129, V185, P186, L187, T188, W190, H191, H218, I243. | Lysate heat-treated for 2 h at 65 °C. *Gf*-PC disc incubated at 65 °C for 24 h with 150 *µ*L clarified lysate (300 *µ*L total volume), 1900 clones screened. | S100L, D98P  (Figure S15) | S100L  (PC-1) |
| 2 | Saturation mutagenesis of positions adjacent to beneficial mutations from Round 1, library positions with high beneficial diversity from Round 1 library screen, and significant residues identified by Tournier *et.al.*^9^: A97, D98, A99, S101, L102, A103, W104, G127, P128, D129, S130, W190, H191, T192, A209, Q217, H218, A219, F222, I243, A251, A281. | Lysate heat-treated for 2 h at 65 °C. *Gf*-PC disc incubated at 65 °C for 2 h with 135 *µ*L clarified lysate (300 *µ*L total volume), 1900 clones screened. | W104L, W104M, W104R, G127F, A209F, A209W  (Figure S15) | G127F, A209F  (PC-2) |

**Table S2:** Primer sequences used to generate DNA libraries. The forward (F) and reverse (R) library primers were paired with the opposite flanking primer to create two gene fragments, which when joined together, formed the mutated gene. Bases which bind to the gene are upper case, with overhangs in lower case. Randomized codon positions are highlighted in red, where N = A, C, T, or G, and K = T or G

| **Flanking primers (Round 1)** | |
| --- | --- |
| GG_F | atactacggtctcaaggaAGCAACCCGTACCAG |
| GG_R | gcattacggtctcgggaaccTTGGCAGTGGCGATT |
| **Round 1** | |
| Y95_F | GCGATGAGTCCGGGTNNKACCGCAGATGCTAGCT |
| Y95_R | ACCCGGACTCATCG |
| A97_F | AGTCCGGGTTATACCNNKGATGCTAGCTCTCTGGC |
| A97_R | GGTATAACCCGGACTCA |
| D98_F | CCGGGTTATACCGCANNKGCTAGCTCTCTGGCAT |
| D98_R | TGCGGTATAACCCGG |
| S100_F | TATACCGCAGATGCTNNKTCTCTGGCATGGCTG |
| S100_R | AGCATCTGCGGTATAACC |
| S101_F | ACCGCAGATGCTAGCNNKCTGGCATGGCTGG |
| S101_R | GCTAGCATCTGCGGT |
| L102_F | GCAGATGCTAGCTCTNNKGCATGGCTGGGTC |
| L102_R | AGAGCTAGCATCTGCG |
| A103_F | GATGCTAGCTCTCTGNNKTGGCTGGGTCGTC |
| A103_R | CAGAGAGCTAGCATCTGC |
| R107_F | CTGGCATGGCTGGGTNNKCGCCTGGCTTCC |
| R107_R | ACCCAGCCATGCCA |
| P128_F | TCACGTTTCGATGGCNNKGACAGCCGCGC |
| P128_R | GCCATCGAAACGTGAAT |
| D129_F | CGTTTCGATGGCCCGNNKAGCCGCGCCTCT |
| D129_R | CGGGCCATCGAAAC |
| H164_F | CTGGCGGTTGCCGGTNNKTCTATGGGTGGCGG |
| H164_R | ACCGGCAACCGC |
| M166_F | GTTGCCGGTCATTCTNNKGGTGGCGGTGGCA |
| M166_R | AGAATGACCGGCAACC |
| V185_F | AGCCTGAAAGCGGCTNNKCCGCTGACCCCGT |
| V185_R | AGCCGCTTTCAGGC |
| P186_F | CTGAAAGCGGCTGTCNNKCTGACCCCGTGGC |
| P186_R | GACAGCCGCTTTCAG |
| L187_F | AAAGCGGCTGTCCCGNNKACCCCGTGGCAC |
| L187_R | CGGGACAGCCGCTTT |
| T188_F | GCGGCTGTCCCGCTGNNKCCGTGGCACACCGATAAA |
| T188_R | CAGCGGGACAGCC |
| W190_F | GTCCCGCTGACCCCGNNKCACACCGATAAAACGTTTAATACC |
| W190_R | CGGGGTCAGCGG |
| H191_F | CCGCTGACCCCGTGGNNKACCGATAAAACGTTTAATACCAGT |
| H191_R | CCACGGGGTCAGC |
| V212_F | GCAGAAGCTGACACCNNKGCGCCGGTTTCG |
| V212_R | GGTGTCAGCTTCTGC |
| H218_F | GCGCCGGTTTCGCAGNNKGCCATCCCGTTTTATCAAAAC |
| H218_R | CTGCGAAACCGGC |
| I243_F | ACGACGACGACGACGNNKGCTCCGAATAGCAACAATG |
| I243_R | GTGCGATGCGTTGC |
| N246_F | TCGCACATTGCTCCGNNKAGCAACAATGCGGC |
| N246_R | CGGAGCAATGTGCGA |

| **Round 2** | |
| --- | --- |
| A97_F | AGTCCGGGTTATACCNNKGATGCTCTTTCTCTGGCA |
| A97_R | GGTATAACCCGGACTCA |
| D98_F | CCGGGTTATACCGCANNKGCTCTTTCTCTGGCATG |
| D98_R | TGCGGTATAACCCGG |
| A99_F | CCGGGTTATACCGCANNKGCTCTTTCTCTGGCATG |
| A99_R | ATCTGCGGTATAACCCG |
| S101_F | CCGGGTTATACCGCANNKGCTCTTTCTCTGGCATG |
| S101_R | AAGAGCATCTGCGGTATA |
| L102_F | GCAGATGCTCTTTCTNNKGCATGGCTGGGTCGT |
| L102_R | AGAAAGAGCATCTGCGG |
| A103_F | GCAGATGCTCTTTCTNNKGCATGGCTGGGTCGT |
| A103_R | CAGAGAAAGAGCATCTGC |
| W104_F | GCTCTTTCTCTGGCANNKCTGGGTCGTCGCC |
| W104_R | TGCCAGAGAAAGAGCAT |
| G127_F | AATTCACGTTTCGATNNKCCGGACAGCCGC |
| G127_R | ATCGAAACGTGAATTCGTGT |
| P128_F | TCACGTTTCGATGGCNNKGACAGCCGCGCCT |
| P128_R | GCCATCGAAACGTGAATTC |
| D129_F | CGTTTCGATGGCCCGNNKAGCCGCGCCTCT |
| D129_R | CGGGCCATCGAAAC |
| S130_F | TCACGTTTCGATGGCNNKGACAGCCGCGCCT |
| S130_R | GTCCGGGCCATCGAA |
| W190_F | GTCCCGCTGACCCCGNNKCACACCGATAAAACGTTTAATACC |
| W190_R | CGGGGTCAGCGG |
| H191_F | CCGCTGACCCCGTGGNNKACCGATAAAACGTTTAATACCAGT |
| H191_R | CCACGGGGTCAGC |
| T192_F | CTGACCCCGTGGCACNNKGATAAAACGTTTAATACCAGTGTCC |
| T192_R | GTGCCACGGGGT |
| A209_F | ATTGTTGGCGCAGAANNKGACACCGTGGCGC |
| A209_R | TTCTGCGCCAACAATC |
| Q217_F | GTGGCGCCGGTTTCGNNKCATGCCATCCCGTTTTATC |
| Q217_R | CGAAACCGGCGC |
| H218_F | GCGCCGGTTTCGCAGNNKGCCATCCCGTTTTATCAAAAC |
| H218_R | CTGCGAAACCGGC |
| A219_F | CCGGTTTCGCAGCATNNKATCCCGTTTTATCAAAACCTG |
| A219_R | ATGCTGCGAAACCGG |
| F222_F | CAGCATGCCATCCCGNNKTATCAAAACCTGCCGAGC |
| F222_R | CGGGATGGCATGCT |
| I243_F | ACGACGACGACGACGNNKGCTCCGAATAGCAACAATG |
| I243_R | GTGCGATGCGTTGC |
| A251_F | AATAGCAACAATGCGNNKATTTCCGTTTATACGATCTCATGG |
| A251_R | CGCATTGTTGCTATTCGG |
| A281_F | AATGTGAACGACCCGNNKCTGTGCGACTTCCGC |
| A281_R | CGGGTCGTTCACATTAC |

# **DNA and Protein Sequences**

**pET26_LCC^ICCG^ nucleotide sequence:**

ATGTCAGGAAGCAACCCGTACCAGCGTGGCCCGAATCCGACCCGCAGCGCACTGACCGCAGATGGCCCGTTTAGCGTGGCAACCTACACCGTCTCACGCCTGTCAGTCTCGGGTTTTGGCGGTGGCGTGATTTATTACCCGACCGGCACGTCTCTGACGTTCGGTGGCATCGCGATGAGTCCGGGTTATACCGCAGATGCTAGCTCTCTGGCATGGCTGGGTCGTCGCCTGGCTTCCCATGGCTTTGTGGTTCTGGTGATTAACACGAATTCACGTTTCGATGGCCCGGACAGCCGCGCCTCTCAGCTGAGTGCCGCCCTGAACTACCTGCGTACCAGTTCCCCGAGCGCCGTTCGCGCACGTCTGGATGCAAATCGTCTGGCGGTTGCCGGTCATTCTATGGGTGGCGGTGGCACCCTGCGTATTGCAGAACAAAACCCGAGCCTGAAAGCGGCTGTCCCGCTGACCCCGTGGCACACCGATAAAACGTTTAATACCAGTGTCCCGGTGCTGATTGTTGGCGCAGAAGCTGACACCGTGGCGCCGGTTTCGCAGCATGCCATCCCGTTTTATCAAAACCTGCCGAGCACCACGCCGAAAGTTTACGTCGAACTGTGCAACGCATCGCACATTGCTCCGAATAGCAACAATGCGGCCATTTCCGTTTATACGATCTCATGGATGAAACTGTGGGTCGATAATGACACCCGTTACCGCCAGTTCCTGTGTAATGTGAACGACCCGGCTCTGTGCGACTTCCGCACCAATAATCGCCACTGCCAAGGTTCCCTCGAGCACCACCACCACCACCAC

**pET26_LCC^ICCG^ expressed amino acid sequence (amino acid numbering starts at 33):**

M^33^SGSNPYQRGPNPTRSALTADGPFSVATYTVSRLSVSGFGGGVIYYPTGTSLTFGGIAMSPGYTADASSLAWLGRRLASHGFVVLVINTNSRFDGPDSRASQLSAALNYLRTSSPSAVRARLDANRLAVAGHSMGGGGTLRIAEQNPSLKAAVPLTPWHTDKTFNTSVPVLIVGAEADTVAPVSQHAIPFYQNLPSTTPKVYVELCNASHIAPNSNNAAISVYTISWMKLWVDNDTRYRQFLCNVNDPALCDFRTNNRHCQGSLEHHHHHH

**pET26_PC-2 nucleotide sequence:**

ATGTCAGGAAGCAACCCGTACCAGCGTGGCCCGAATCCGACCCGCAGCGCACTGACCGCAGATGGCCCGTTTAGCGTGGCAACCTACACCGTCTCACGCCTGTCAGTCTCGGGTTTTGGCGGTGGCGTGATTTATTACCCGACCGGCACGTCTCTGACGTTCGGTGGCATCGCGATGAGTCCGGGTTATACCGCAGATGCTCTTTCTCTGGCATGGCTGGGTCGTCGCCTGGCTTCCCATGGCTTTGTGGTTCTGGTGATTAACACGAATTCACGTTTCGATTTTCCGGACAGCCGCGCCTCTCAGCTGAGTGCCGCCCTGAACTACCTGCGTACCAGTTCCCCGAGCGCCGTTCGCGCACGTCTGGATGCAAATCGTCTGGCGGTTGCCGGTCATTCTATGGGTGGCGGTGGCACCCTGCGTATTGCAGAACAAAACCCGAGCCTGAAAGCGGCTGTCCCGCTGACCCCGTGGCACACCGATAAAACGTTTAATACCAGTGTCCCGGTGCTGATTGTTGGCGCAGAATTTGACACCGTGGCGCCGGTTTCGCAGCATGCCATCCCGTTTTATCAAAACCTGCCGAGCACCACGCCGAAAGTTTACGTCGAACTGTGCAACGCATCGCACATTGCTCCGAATAGCAACAATGCGGCCATTTCCGTTTATACGATCTCATGGATGAAACTGTGGGTCGATAATGACACCCGTTACCGCCAGTTCCTGTGTAATGTGAACGACCCGGCTCTGTGCGACTTCCGCACCAATAATCGCCACTGCCAAGGTTCCCTCGAGCACCACCACCACCACCAC

**pET26_PC-2 expressed amino acid sequence (amino acid numbering starts at 33):**

M^33^SGSNPYQRGPNPTRSALTADGPFSVATYTVSRLSVSGFGGGVIYYPTGTSLTFGGIAMSPGYTADALSLAWLGRRLASHGFVVLVINTNSRFDFPDSRASQLSAALNYLRTSSPSAVRARLDANRLAVAGHSMGGGGTLRIAEQNPSLKAAVPLTPWHTDKTFNTSVPVLIVGAEFDTVAPVSQHAIPFYQNLPSTTPKVYVELCNASHIAPNSNNAAISVYTISWMKLWVDNDTRYRQFLCNVNDPALCDFRTNNRHCQGSLEHHHHHH

# **References**

(1) Son, H. F.; Cho, I. J.; Joo, S.; Seo, H.; Sagong, H.-Y.; Choi, S. Y.; Lee, S. Y.; Kim, K.-J. "Rational Protein Engineering of Thermo-Stable PETase from Ideonella Sakaiensis for Highly Efficient PET Degradation." *ACS Catal.* **2019**, *9*, 3519–3526. DOI: 10.1021/acscatal.9b00568

(2) Bell, E. L.; Smithson, R.; Kilbride, S.; Foster, J.; Hardy, F. J.; Ramachandran, S.; Tedstone, A. A.; Haigh, S. J.; Garforth, A. A.; Day, P. J. R.; Levy, C.; Shaver, M. P.; Green, A. P. "Directed Evolution of an Efficient and Thermostable PET Depolymerase." *Nat. Catal.* **2022**, *5*, 673–681. DOI: 10.1038/s41929-022-00821-3

(3) Then, J.; Wei, R.; Oeser, T.; Barth, M.; Belisário-Ferrari, M. R.; Schmidt, J.; Zimmermann, W. "Ca2+ and Mg2+ Binding Site Engineering Increases the Degradation of Polyethylene Terephthalate Films by Polyester Hydrolases from *Thermobifida Fusca*." *Biotechnol. J.* **2015**, *10*, 592–598. DOI: 10.1002/biot.201400620

(4) Ufarté, L.; Laville, E.; Duquesne, S.; Morgavi, D.; Robe, P.; Klopp, C.; Rizzo, A.; Pizzut-Serin, S.; Potocki-Veronese, G. "Discovery of Carbamate Degrading Enzymes by Functional Metagenomics." *PLoS One* **2017**, *12*, 1–21. DOI: 10.1371/journal.pone.0189201

(5) Anderson, J. C.; Dueber, J. E.; Leguia, M.; Wu, G. C.; Goler, J. A.; Arkin, A. P.; Keasling, J. D. "BglBricks: A Flexible Standard for Biological Part Assembly." *J. Biol. Eng.* **2010**, *4*, 1. DOI: 10.1186/1754-1611-4-1

(6) Ronkvist, Å. M.; Xie, W.; Lu, W.; Gross, R. A. "Cutinase-Catalyzed Hydrolysis of Poly(Ethylene Terephthalate)." *Macromolecules* **2009**, *42*, 5128–5138. DOI: 10.1021/ma9005318

(7) Kawabata, T.; Oda, M.; Kawai, F. "Mutational Analysis of Cutinase-like Enzyme, Cut190, Based on the 3D Docking Structure with Model Compounds of Polyethylene Terephthalate." *J. Biosci. Bioeng.* **2017**, *124*, 28–35. DOI: 10.1016/j.jbiosc.2017.02.007

(8) Sulaiman, S.; Yamato, S.; Kanaya, E.; Kim, J.-J.; Koga, Y.; Takano, K.; Kanaya, S. "Isolation of a Novel Cutinase Homolog with Polyethylene Terephthalate-Degrading Activity from Leaf-Branch Compost by Using a Metagenomic Approach." *Appl. Environ. Microbiol.* **2012**, *78*, 1556–1562. DOI: 10.1128/AEM.06725-11

(9) Tournier, V.; Topham, C. M.; Gilles, A.; David, B.; Folgoas, C.; Moya-Leclair, E.; Kamionka, E.; Desrousseaux, M. L.; Texier, H.; Gavalda, S.; Cot, M.; Guémard, E.; Dalibey, M.; Nomme, J.; Cioci, G.; Barbe, S.; Chateau, M.; André, I.; Duquesne, S.; Marty, A. "An Engineered PET Depolymerase to Break down and Recycle Plastic Bottles." *Nature* **2020**, *580*, 216–219. DOI: 10.1038/s41586-020-2149-4

(10) Pfaff, L.; Gao, J.; Li, Z.; Jäckering, A.; Weber, G.; Mican, J.; Chen, Y.; Dong, W.; Han, X.; Feiler, C. G.; Ao, Y. F.; Badenhorst, C. P. S.; Bednar, D.; Palm, G. J.; Lammers, M.; Damborsky, J.; Strodel, B.; Liu, W.; Bornscheuer, U. T.; Wei, R. "Multiple Substrate Binding Mode-Guided Engineering of a Thermophilic PET Hydrolase." *ACS Catal.* **2022**, *12*, 9790–9800. DOI: 10.1021/acscatal.2c02275

(11) Lu, H.; Diaz, D. J.; Czarnecki, N. J.; Zhu, C.; Kim, W.; Shroff, R.; Acosta, D. J.; Alexander, B. R.; Cole, H. O.; Zhang, Y.; Lynd, N. A.; Ellington, A. D.; Alper, H. S. "Machine Learning-Aided Engineering of Hydrolases for PET Depolymerization." *Nature* **2022**, *604*, 662–667. DOI: 10.1038/s41586-022-04599-z

(12) Cui, Y.; Chen, Y.; Liu, X.; Dong, S.; Tian, Y.; Qiao, Y.; Mitra, R.; Han, J.; Li, C.; Han, X.; Liu, W.; Chen, Q.; Wei, W.; Wang, X.; Du, W.; Tang, S.; Xiang, H.; Liu, H.; Liang, Y.; Houk, K. N.; Wu, B. "Computational Redesign of a PETase for Plastic Biodegradation under Ambient Condition by the GRAPE Strategy." *ACS Catal.* **2021**, *11*, 1340–1350. DOI: 10.1021/acscatal.0c05126

(13) Holst, L. H.; Madsen, N. G.; Toftgård, F. T.; Rønne, F.; Moise, I.-M.; Petersen, E. I.; Fojan, P. "De Novo Design of a Polycarbonate Hydrolase." *Protein Eng. Des. Sel.* **2023**, *36*, 1–11. DOI: 10.1093/protein/gzad022

(14) Bird, J. E.; Marles-Wright, J.; Giachino, A. "A User’s Guide to Golden Gate Cloning Methods and Standards." *ACS Synth. Biol.* **2022**, *11*, 3551–3563. DOI: 10.1021/acssynbio.2c00355

(15) Studier, F. W. "Protein Production by Auto-Induction in High-Density Shaking Cultures." *Protein Expr. Purif.* **2005**, *41*, 207–234. DOI:10.1016/j.pep.2005.01.016

(16) Abraham, M. J.; Murtola, T.; Schulz, R.; Páll, S.; Smith, J. C.; Hess, B.; Lindahl, E. "GROMACS: High performance molecular simulations through multi-level parallelism from laptops to supercomputers." *SoftwareX* **2015**, *1*, 19-25.

(17) Pall, S.; Abraham, M. J.; Kutzner, C.; Hess, B.; Lindahl, E. "Tackling Exascale Software Challenges in Molecular Dynamics Simulations with GROMACS." In *2nd International Conference on Exascale Applications and Software (EASC)*, Stockholm, SWEDEN, Apr 02-03, 2014; 2015; Vol. 8759, pp 3-27. DOI: 10.1007/978-3-319-15976-8_1.

(18) Maier, J. A.; Martinez, C.; Kasavajhala, K.; Wickstrom, L.; Hauser, K. E.; Simmerling, C. "ff14SB: improving the accuracy of protein side chain and backbone parameters from ff99SB." *Journal of chemical theory and computation* **2015**, *11* (8), 3696-3713.

(19) Bussi, G.; Donadio, D.; Parrinello, M. "Canonical sampling through velocity rescaling." *The Journal of chemical physics* **2007**, *126* (1), 014101.

(20) Nosé, S.; Klein, M. "Constant pressure molecular dynamics for molecular systems." *Molecular Physics* **1983**, *50* (5), 1055-1076.

(21) Hess, B.; Bekker, H.; Berendsen, H. J.; Fraaije, J. G. "LINCS: a linear constraint solver for molecular simulations." *Journal of computational chemistry* **1997**, *18* (12), 1463-1472.

(22) Bottegoni, G.; Kufareva, I.; Totrov, M.; Abagyan, R.**"**Four-dimensional docking: a fast and accurate account of receptor flexibility." *Journal of Computational Chemistry* **2009,** 30 (13), 2056-2067.
